# A general framework for functionally informed set-based analysis: Application to a large-scale colorectal cancer study

**DOI:** 10.1371/journal.pgen.1008947

**Published:** 2020-08-24

**Authors:** Xinyuan Dong, Yu-Ru Su, Richard Barfield, Stephanie A. Bien, Qianchuan He, Tabitha A. Harrison, Jeroen R. Huyghe, Temitope O. Keku, Noralane M. Lindor, Clemens Schafmayer, Andrew T. Chan, Stephen B. Gruber, Mark A. Jenkins, Charles Kooperberg, Ulrike Peters, Li Hsu

**Affiliations:** 1 Division of Public Health Sciences, Fred Hutchinson Cancer Research Center, Seattle, WA, USA; 2 Department of Biostatistics, University of Washington, Seattle, WA, USA; 3 Center for Gastrointestinal Biology and Disease, University of North Carolina, Chapel Hill, North Carolina, USA; 4 Department of Health Science Research, Mayo Clinic, Scottsdale, Arizona, USA; 5 Department of General Surgery, University Hospital Rostock, Rostock, Germany; 6 Division of Gastroenterology, Massachusetts General Hospital and Harvard Medical School, and Channing Division of Network Medicine, Brigham and Women’s Hospital and Harvard Medical School, Boston, Massachusetts, USA; 7 City of Hope National Medical Center, Duarte, and Department of Preventive Medicine & USC Norris Comprehensive Cancer Center, Keck School of Medicine, University of Southern California, Los Angeles, California, USA; 8 Centre for Epidemiology and Biostatistics, Melbourne School of Population and Global Health, University of Melbourne, Melbourne, Victoria, Australia; University College London, UNITED KINGDOM

## Abstract

Genome-wide association studies (GWAS) have successfully identified tens of thousands of genetic variants associated with various phenotypes, but together they explain only a fraction of heritability, suggesting many variants have yet to be discovered. Recently it has been recognized that incorporating functional information of genetic variants can improve power for identifying novel loci. For example, S-PrediXcan and TWAS tested the association of predicted gene expression with phenotypes based on GWAS summary statistics by leveraging the information on genetic regulation of gene expression and found many novel loci. However, as genetic variants may have effects on more than one gene and through different mechanisms, these methods likely only capture part of the total effects of these variants. In this paper, we propose a summary statistics-based mixed effects score test (sMiST) that tests for the total effect of both the effect of the mediator by imputing genetically predicted gene expression, like S-PrediXcan and TWAS, and the direct effects of individual variants. It allows for multiple functional annotations and multiple genetically predicted mediators. It can also perform conditional association analysis while adjusting for other genetic variants (e.g., known loci for the phenotype). Extensive simulation and real data analyses demonstrate that sMiST yields p-values that agree well with those obtained from individual level data but with substantively improved computational speed. Importantly, a broad application of sMiST to GWAS is possible, as only summary statistics of genetic variant associations are required. We apply sMiST to a large-scale GWAS of colorectal cancer using summary statistics from ∼120, 000 study participants and gene expression data from the Genotype-Tissue Expression (GTEx) project. We identify several novel and secondary independent genetic loci.

## Author summary

We developed summary statistics-based mixed effects score test statistics (sMiST) for testing the association of multiple genetically predicted mediators simultaneously and direct association of individual variants independent of mediators by using a random effects model. Extensive simulation and real data analyses demonstrate that sMiST recovers the results of MiST that is based on individual level data, but is computationally much faster. We applied our approach to a genome-wide association study of colorectal cancer and gene expression and identified several novel and secondary genetic loci.

## Introduction

Single variant analysis in genome-wide association studies (GWAS) has been successful in identifying thousands of variants associated with various diseases and traits [1]. However, these variants all together explain only a fraction of heritability, suggesting that many variants remain to be discovered. Until now, most of these discoveries have been mainly driven by increases in sample size. The gain from substantially increasing sample size is diminishing, but incorporation of functional knowledge about the genome will likely play a critical role in informing discovery of novel loci as well as understanding the pathways in which the genetic loci may be involved.

Research for integrating functional knowledge into GWAS has been active recently. This is in part due to success of large collaborative projects such as the Genotype-Tissue Expression (GTEx) project [2] and the Encyclopedia of DNA Elements (ENCODE) [3], which have generated extensive knowledge about functions of genetic variants that can be used for aggregating and weighting genetic variants. Widely available GWAS summary statistics for individual variants has made it possible to leverage these functional information, leading to many more discoveries of novel genetic loci. For example, PrediXcan [4, 5] and TWAS [6] test the association between genetically predicted gene expression levels and phenotypes. A comprehensive review and comparison of various methods can be found in Barbeira et al. (2018) [5]. The TWAS-like analysis can also be framed as a class of Mendelian randomization [7, 8], in which under some assumptions the mediator effect of gene expression can be estimated by the inverse variance weighted ratios of regression coefficients of genetic variants for the phenotype and those for the gene expression. All of these methods also apply to other types of mediators including methylation and lifestyle variables (e.g., smoking) that may be regulated by genetic variants.

These approaches could be considered as a type of set-based association test, in which the predictor is the weighted sum of a set of genetic variants with weights being the effect sizes on gene expression. However, these methods do not take into account the potential effects of genetic variants beyond their effects on expression of a specific gene. Complex trait loci typically map to regions of the genome clustered with regulatory elements, which in turn have combinatorial effects on the expression of several target genes [9, 10]. Variants may have functional effects on more than one gene through their disruption of multiple regulatory elements [9]. Consequently, these approaches likely only capture part of the total effect of expression quantitative trait loci (eQTL).

We recently proposed a Mixed effects Score Test (MiST), which formulates the association of mediators (fixed effects), while allowing for effects of individual variants on disease risk directly adjusting for mediators (random effects) [11, 12]. Thus, MiST can increase power if some variants individually influence disease risk through other functional mechanisms besides mediators (e.g., gene expression). Another advantage of MiST is that the test statistics for the mediation and direct effects are independent, providing a flexible framework for optimally combining the two components to achieve the maximal power. However, the test statistics of MiST were derived from individual-level data, which cannot be applied if there is difficulty in accessing individual-level data. To maximize power for detecting novel genetic association, it is desired to conduct association analysis using GWAS summary statistics, facilitating pooling data across studies and consortia.

In this paper, we propose forming the test statistics of MiST based on summary statistics, which we term as sMiST. There are several novel contributions: 1) simultaneous testing of multiple mediators (e.g., gene expression and methylation); 2) testing of the variance component of the direct effects of genetic variants independent of the mediation effects; 3) combining the test statistics of both mediation and direct effects to form a single overall test that can capture information from both mediation and direct effects; 4) conditional testing of mediation and direct effects, adjusting for multiple other genetics variants. For example, one may perform conditional testing to examine whether a finding is novel conditional on known loci. When there is only one mediator, the test statistic for testing the association of the mediator under the assumption of no direct effects has the same form as PredXcan and TWAS [5, 6] and the Mendelian randomization two-stage estimator [8, 13]. Our method also avoids the direct inversion of the covariance matrix of genotypes as in the Mendelian randomization approach for dealing with correlated variants [8], therefore, it can work on genes with varying degrees of correlation structures, without any variant pruning. We show that our method of combining summary statistics gives p-values that are consistent with those constructed from individual level data, and that it is much more efficient computationally. We applied sMiST to a large-scale GWAS of colorectal cancer using summary statistics, and identified three novel loci that are located 1MB outside of known CRC loci regions, as well as one additional secondary novel locus.

## Methods

### MiST framework

Consider an outcome *Y*, which can be continuous or binary. We are interested in the association of a set of *P* variants *G* = (*G*_1_, …, *G*_*P*_) with outcome *Y*. Assume there are *K* mediating variables *M* = (*M*_1_, *M*_2_, …, *M*_*K*_)^*T*^, with which *G* are associated and *K* < *P*; here the superscript *T* is for transpose. Let *X* be a vector of confounders including the intercept. The confounders may include study, age, sex, and principle components to account for population structure in the data. A generalized linear model can be used to assess the association of *M* and *G* while adjusting for confounders *X*

$$\begin{array}{ccc} g\{\text{E}(Y|X,M,G)\} & = & X^{T}\eta +\sum_{k=1}^{K}M_{k}\gamma_{k}+\sum_{p=1}^{P}\delta_{p}G_{p}, \end{array}$$ (1)

where *g*(⋅) is a logit function if *Y* is binary and an identity function if *Y* is continuous. The regression coefficients *η*, *γ* = (*γ*_1_, …, *γ*_*K*_)^*T*^, and *δ* = (*δ*_1_, …, *δ*_*P*_)^*T*^ are the effects of the confounders, *K* mediators, and direct effects of *G*, respectively. To obtain estimators for *γ* and *δ*, ideally the measurements of genotypes *G*, mediators *M*, and outcome *Y* are collected on the same set of individuals. However, GWAS usually have very large sample sizes because of their need to detect modest genetic effects; as such, collecting mediators *M* such as gene expression and methylation on all individuals in GWAS can be costly and logistically difficult.

As *M* is not measured, it is instructive to examine *E*(*Y*|*X*, *G*) by integrating out the unmeasured *M* under the true model (1) [14]. If *g* is linear, it is straightforward to see that $\text{E}(Y|X,G)=X^{T}\eta +\sum_{k=1}^{K}\text{E}\left(M_{k}|G,X\right)\gamma_{k}+\sum_{p=1}^{P}\delta_{p}G_{p}$. This suggests that if there is a model that can predict *M*_*k*_ well using *G*, we can use this model to impute the missing *M*_*k*_ by *E*(*M*_*k*_|*G*, *X*). If *g* is logit, there is no closed form for *E*(*Y*|*X*, *G*) but we can approximate $g\left\{\text{E}\left(Y|X,G\right)\right\}\approx \left\{X\eta +\text{E}\left(M|G,X\right)\gamma +\sum_{p=1}^{P}\delta_{p}G_{p}\right\}/\phi$, where *ϕ* = {1+ *γ*^*T*^ *cov*(*M*|*G*, *X*)*γ*/1.7^2^}^1/2^ [15]. Despite that the parameters are attenuated by 1/*ϕ* under this model, testing of null association for the mediation with *E*(*M*|*G*, *X*) and direct effects with {*G*_*p*_, *p* = 1, …, *P*} is equivalent to testing *γ* and *δ*’s = 0 in model (1). For simplicity, we used the same notation {*γ*, *δ*} for the attenuated parameters in the following models.

Specifically, we fit a linear regression model to the mediators:

$$\begin{array}{ccc} \text{E}(M_{k}|X,G) & = & X^{T}\eta +\sum_{p=1}^{P}W_{pk}G_{p},\;k=1,\ldots ,K, \end{array}$$ (2)

where *W*_*pk*_ is the weight or regression coefficient of *p*th variant associated with the *k*th mediator. Here, to avoid introducing too many non-critical notation, we use *η* to denote generically the regression coefficients for confounders *X* and they may not be same as *η* for confounders in other models. The weight *W*_*pk*_ is set to 0 if the *p*th variant is not associated with *k*th mediator. In some situations, some variants that are associated with mediators *M* are not part of the set {*G*_*p*_, *p* = 1, …, *P*}. We can expand {*G*_*p*_, *p* = 1, …, *P*} to include these variants but set the corresponding *δ*’s in model (1) to 0. Now plugging (2) into (1), we obtain

$$\begin{array}{ccc} g\{\text{E}(Y|X,M,G)\} & = & X^{T}\eta +\sum_{k=1}^{K}{\hat{M}}_{k}\gamma_{k}+\sum_{p=1}^{P}\delta_{p}G_{p}, \end{array}$$ (3)

where ${\hat{M}}_{k}=\sum_{p=1}^{P}W_{pk}G_{p}$ for *k* = 1, …, *K*. To obtain the weights {*W*_*pk*_, *p* = 1, …, *P*, *k* = 1, …*K*}, we can use a reference dataset that has both genotyping and mediator data. The dataset needs not overlap with the GWAS data. For example, PrediXcan uses genetic variants and gene expression data from GTEx and other studies to build a genetically predicted gene expression linear regression model for each gene [4].

As the number of mediators *K* is typically small, we assume *γ* as fixed effects. On the other hand, for testing the direct effects, the number of genetic variants *P* can be large. An omnibus *χ*^2^ test with *P* degrees of freedom may not be powerful. Instead, we assume that *δ*_*p*_, *p* = 1, …, *P*, follow an arbitrary distribution with mean 0 and variance *τ*^2^. Thus, we test the overall null

$$\begin{array}{c} H_{0}:\gamma =0\;\text{and}\;\tau^{2}=0. \end{array}$$

If the test rejects the null hypothesis at a pre-specified significance level, it suggests there is evidence against the null total effects of genetic variants *G* on *Y*. We note that model (3) can also be formulated by a hierarchical model as shown in Su et al. (2018) [12], where in a generalized linear model of G and Y, the main effects of *G* are further modeled by incorporating the functional information of the genetic variants such as weights in predicting gene expression.

### Summary statistics-based mixed effects score test (sMiST)

Su et al. [12] proposed the Mixed effects Score Test (MiST) to test nullity of the fixed effects *γ* and variance component *τ*^2^ using individual level data. Here we introduce a method that requires only summary statistics to perform the tests for *H*_0_: *γ* = 0 and *τ*^2^ = 0. Following the convention, assume we have the summary statistics at hand:

- Standard GWAS output: marginal regression coefficients $\{{\hat{\beta }}_{p}^{*},\text{se}({\hat{\beta }}_{p}^{*}),p=1,2,\ldots ,P\}$ from
  $$\begin{array}{c} g\left\{E\left(Y|X,G_{p}\right)\right\}=X\eta +G_{p}\beta_{p}^{*},\;p=1,\ldots ,P, \end{array}$$
- Covariance of the genotypes, cov(*G*).

The summary statistics can also be replaced by score statistic ${\tilde{U}}_{p}$ and variance ${\tilde{V}}_{p}$ with ${\hat{\beta }}_{p}^{*}\approx {\tilde{V}}_{p}^{-1}{\tilde{U}}_{p}$ and $\text{se}\left({\hat{\beta }}_{p}^{*}\right)\approx {\tilde{V}}_{p}^{-1/2}$. When the variants are rare or less frequent, score statistics are numerically more reliable than the estimates of marginal regression coefficients, because score statistics are calculated under the null. The covariance cov(*G*) can be obtained from an internal random subset of control samples or an external reference database. In the latter case, the reference data should match as close as possible to the underlying population for the summary statistics to avoid false positives [5].

Based on the summary statistics, we derive the test statistics for the overall mediation effects *γ* = 0, as well as individual mediator effects *γ*_*k*_, *k* = 1, …, *K* under *τ*^2^ = 0. In addition, we derive the test statistics for *τ*^2^ = 0, conditional on $\hat{M}$. By conditioning on $\hat{M}$, the test statistic for *τ*^2^ is independent of the test statistic for *γ* [12]. We can then straightforwardly combine the two test statistics by using the p-value-based Fisher’s or minP combination procedure. Alternatively, we can also use data-driven weighted combination methods as in MiST: optimally weighted linear combination and adaptively weighted linear combination, neither of which requires individual level data. We termed the summary statistics-based combined mixed effects test as sMiST.

In theory, p-values derived from summary statistics and p-values derived from individual-level data are asymptotically equivalent under the null if there are no confounders and the estimate of cov(*G*) is accurate, the latter of which is important especially if the genotype data are from an external dataset that differs from the data that generate the summary statistics.

## Results

### Identifying novel genes associated with CRC risk using sMiST

We analyzed a large GWAS of colorectal cancer (54,454 cases and 64,163 controls) [16]. We considered the mediation effect of gene expression and downloaded the estimates of genetic effects on gene expression from the PredictDB Data Repository (http://predictdb.org/). We controlled the overall type I error at 0.05, allocating 0.04 for genome-wide discovery and 0.01 for conditional analysis to identify novel loci while adjusting for known CRC loci. Specifically, we tested 8,893 genes and used a Bonferroni correction to account for multiple testing, which yields a significance level at the gene level 0.04/8893 = 4.5 × 10^−6^. For the conditional analysis, we set the significance level at the gene level to be 0.01 divided by the number of significant genes from the genome-wide discovery.

A total of 90 genes reached the genome-wide significance level of 4.5 × 10^−6^ using optimally weighted linear combination of sMiST (S1 Table). To evaluate whether these genes are novel for CRC, we performed conditional analysis adjusting for the CRC known loci [16] on the same chromosome using sMiST. We constructed a weight matrix *W*_*Q*+*P*, *Q*+1_ such that the first *Q* columns are 1 on the diagonal corresponding to known loci and 0 everywhere else, and the last column is 0 for the first *Q* rows and weights of *P* variants used in predicting gene expression for the remaining *P* rows. We arranged summary statistics for the *Q* known loci and the *P* variants as a vector. It is straightforward to see that the adjusted p-value for the predicted gene expression conditioning on the known loci is as if each of the known loci were a “mediator”.

After adjusting for the CRC known loci risk, four genes remain significant at 0.01/90 = 1.1×10^−4^ (Table 1), three of which have no known loci within 1Mb of transcription start and end sites of the gene. For all four genes, the main association signal comes from the variance component of the random effects of the SNPs, not from the predicted gene expression. A further examination of marginal association along with eQTL weights shows that variants with the larger weights in predicting gene expression do not have evidence for association (*NT5DC2* and *VPREB3*). For *PLD6* and *ANKRD10*, variants that up-regulate (or down-regulate) gene expression have incosistent direction of association with outcome, yielding non-significant p-values for the predicted gene expression (S1–S4 Figs). The odds ratio estimates are close to 1 and their 95% confidence intervals cover 1 (S2 Table). On the other hand, for these genes, several variants do show association with disease risk, for which the variance component test is powerful to detect when the signals are sparse in a set-based test.

**Table 1:** Novel CRC associated genes and secondary genes.

**Novel genes: 0 known loci within 1Mb**									
**Gene Info**				**Unadjusted P-value** ^1^			**Adjusted P-value** ^1^		
Gene	*R* ^2^	N SNPs	chr	Pred Exp	Var Comp	sMiST	Pred Exp	Var Comp	sMiST^2^
*NT5DC2*	0.35	52	3	0.96	1.92e-06	3.96e-06	0.95	2.03e-06	4.38e-06
*PLD6*	0.25	36	17	0.25	1.42e-06	2.89e-06	0.29	9.12e-07	1.99e-06
*VPREB3*	0.04	12	22	0.99	1.41e-06	3.19e-06	0.99	1.41e-06	3.19e-06
**Novel secondary genes: ≥ 1 known loci within 1Mb**									
**Gene Info**				**Unadjusted P-value** ^1^			**Adjusted P-value** ^1^		
Gene	*R* ^2^	N SNPs	chr	Pred Exp	Var Comp	sMiST	Pred Exp	Var Comp	sMiST
*ANKRD10*	0.06	36	13	0.48	1.13e-06	2.36e-06	0.43	2.39e-05	5.45e-05

^1^ The unadjusted and adjusted p-values are without and with adjusting for the known CRC loci that are on the same chromosome of the gene.

^2^ The column names are as follows. *R*^2^ is the variation of gene expression explained by eQTLs from the PrediXcan model; N SNPs is the number of variants in the gene; chr is the chromosome #; Pred Exp is the p-value for predicted gene expression; Var Comp is the p-value for the variance component; sMiST is the combined p-value of predicted gene expression and variance component tests using optimally weighted linear combination.

Next, we conducted a sequential analysis to explore whether the significance of each identified genes is mainly driven by only one variant or a subset of the variants. We first selected the most marginally significant variant after adjusting for known loci. Then we included known loci and also this most significant variant in sMiST to evaluate the association. If the p-value from either the predicted gene expression or variance component was <0.05, we continued the process and selected the next most significant variant, adjusting for known loci and previously included variants, until neither the predicted gene expression nor variance component p-value reached significance at 0.05. The association of all of these genes is driven by two or more variants (S3 Table). In particular, for gene *ANKRD10*, 8 variants are associated with CRC risk. All of these highlight the power of set-based association testing that incorporates functional information.

### Performance of sMiST in simulation

We evaluated the performance of summary statistics-based sMiST in testing the mediation and variance components. We examined the type I error of sMiST by generating *Y* assuming both *γ* = 0 and *τ*^2^ = 0. The type I error for sMiST as well as for the mediation and variance component tests is well kept (S4 Table). Importantly, we would like to examine how closely sMiST p-values are compared with the p-values from MiST that were calculated based on individual level data, which we treated as the gold standard. This is because an essential property of summary statistics-based test statistics is that they should agree well with the test statistics obtained as if individual level data were available. We selected three different genes due to their different genetic structures. As the performance of sMiST is similar for all three genes, we only present the results for the *CXCR1* gene here, and show the results of the other two genes (*C18orf32* and *ARHGAP11A*) in S5 and S6 Figs, respectively. Gene *CXCR1* has 42 variants with several clusters of high correlation. Details of simulation are provided in Methods and Materials.

#### Impact of confounding

As the asymptotic equivalence between sMiST and individual-level data based MiST holds when there are no confounders, we examined extensively the impact of confounders on sMiST. We calculated cov(*G*) using the same genotyping data as for generating the outcome. The robustness of cov(*G*) estimated from smaller sample size and external data will be assessed in the section of “Performance of sMiST in real data analysis”. For *CXCR1*, there is one known locus outside the gene, which is highly correlated with the predicted gene expression (mediation) with correlation of −0.66. We thus created a confounding variable by summing this known locus and other independent genetic variants weighted by their marginal effect sizes. We varied the number of independent variants added to the confounding variable to yield the correlation between the confounder and predicted gene ranging from 0.1 to 0.4, representing moderate to high correlation. We also varied the effect size of the confounder, *β*, from 0.3 to 0.9 for modest to strong effect.

We considered 4 general scenarios:

1. Complete null,
2. Null mediation effect and non-zero variance component,
3. Non-zero mediation effect and null variance component, and
4. Non-zero for both mediation effect and variance component.

**Fig 1:**
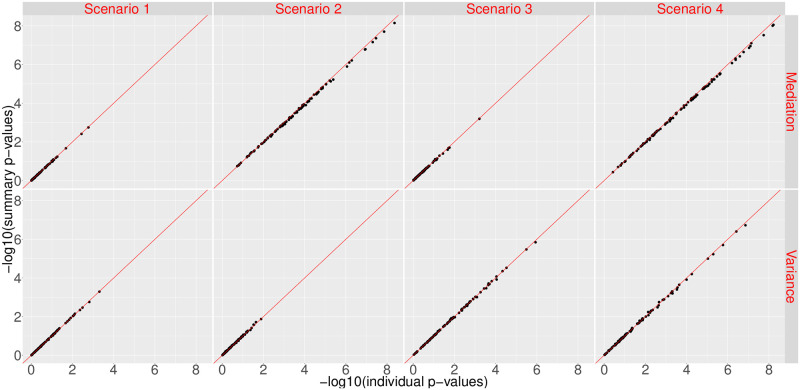
Scatter plots of −log10(p-values) for testing the mediation effect and variance component for sMiST compared with individual level data based MiST in the presence of confounding.

Fig 1 shows the scatter plots of −log10(p-values) for the mediation effect (top) and variance component (bottom) of sMiST and individual-level data based MiST, when the correlation between confounder and predicted gene expression is 0.25, and *β* = 0.6. It is clear that the points fall on the 45 degree line, suggesting sMiST provides virtually identical results to the individual level data based MiST for both mediation and variance components under all four scenarios. In fact, sMiST performs very well compared with MiST even when the correlation is as high as 0.4 and *β* is 0.9 (S7 Fig).

#### Performance of sMiST with multiple mediators

Our method can be generalized to instances when there are multiple mediators. To illustrate, we generated two correlated mediators. One mediator was predicted gene expression of of *CXCR1*, and the other “mediator” is the known CRC locus outside of the gene, which is in nearly perfect correlation with one of the variants in *CXCR1*. This is to mimic the scenario for testing the joint and conditional effect of predicted gene expression and known locus. We combined the genotype data of *CXCR1* and of the known CRC locus into a mega-genotype *n* × (*P* + 1) matrix, where *n* is the number of subjects and *P* is the number of eQTLs in the *CXCR1* gene. We assigned a (*P* + 1) × 2 weight matrix of the form (*W*_1_, *W*_2_), where *W*_1_ = (*w*_1_, …, *w*_*P*_, 0)^*T*^ and *W*_2_ = (0, …, 0, 1)^*T*^. Here the weight is again from the the PredictDB Data Repository.

**Fig 2:**
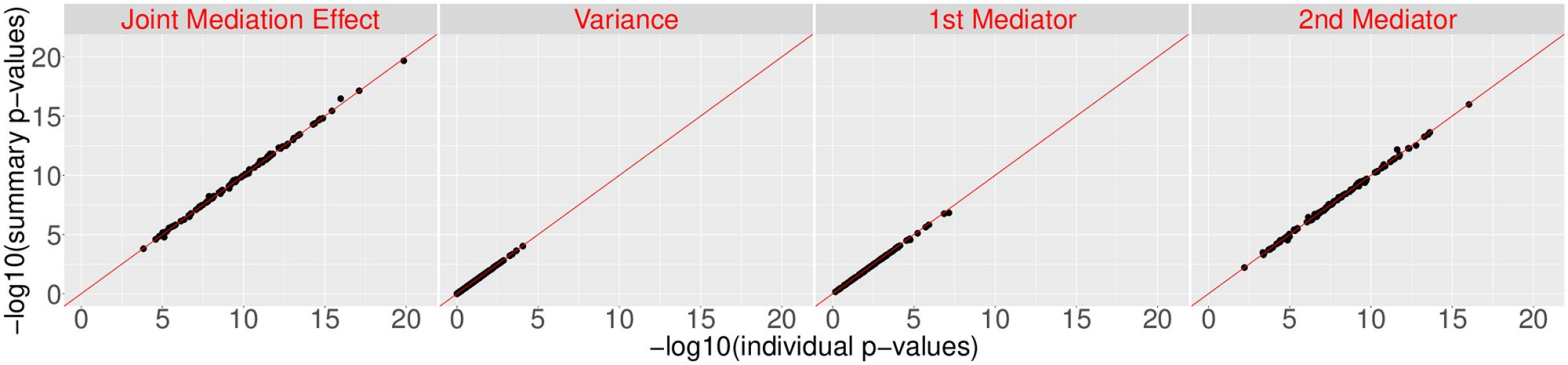
Performance of sMiST when there are two mediators.

We present the p-value for testing the joint mediation effect and the p-value for the variance component, as well as the individual p-values associated with each component. sMiST again shows virtual identical p-values with individual-level data based p-values for both the joint mediation effect and individual mediator’s effect conditional on the other mediator (Fig 2).

### Performance of sMiST with rare variants

We compared the performance of summary statistics based sMiST with individual level data based MiST for rare variants with MAF from 0.1% to 1.0%. We calculated sMiST using $\left\{{\hat{\beta }}_{p}^{*},\text{se}\left({\hat{\beta }}_{p}^{*}\right)\right\}$ denoted by sMiST-Wald and score statistics $\left\{{\tilde{U}}_{p},{\tilde{V}}_{p}\right\}$ denoted by sMiST-Score. We also calculated sMiST using the standardized score test statistics to address the situation that only the z statistics {*Z*_*p*_, *p* = 1, …, *P*} from score or likelihood ratio tests and the directions of the effects are available. In this situation, we replaced ${\hat{\beta }}_{p}^{*}$ by $\text{sign}\left(\beta_{p}^{*}\right)Z_{p}$, se(${\hat{\beta }}_{p}^{*}$) by 1, and the covariance of the genotypes by the correlation of the genotypes, where *sign* is 1 if $\beta_{p}^{*}$ is > 0, -1 if $\beta_{p}^{*}$ is < 0, and 0 otherwise. We denote this by sMiST-Standardized Score.

**Fig 3:**
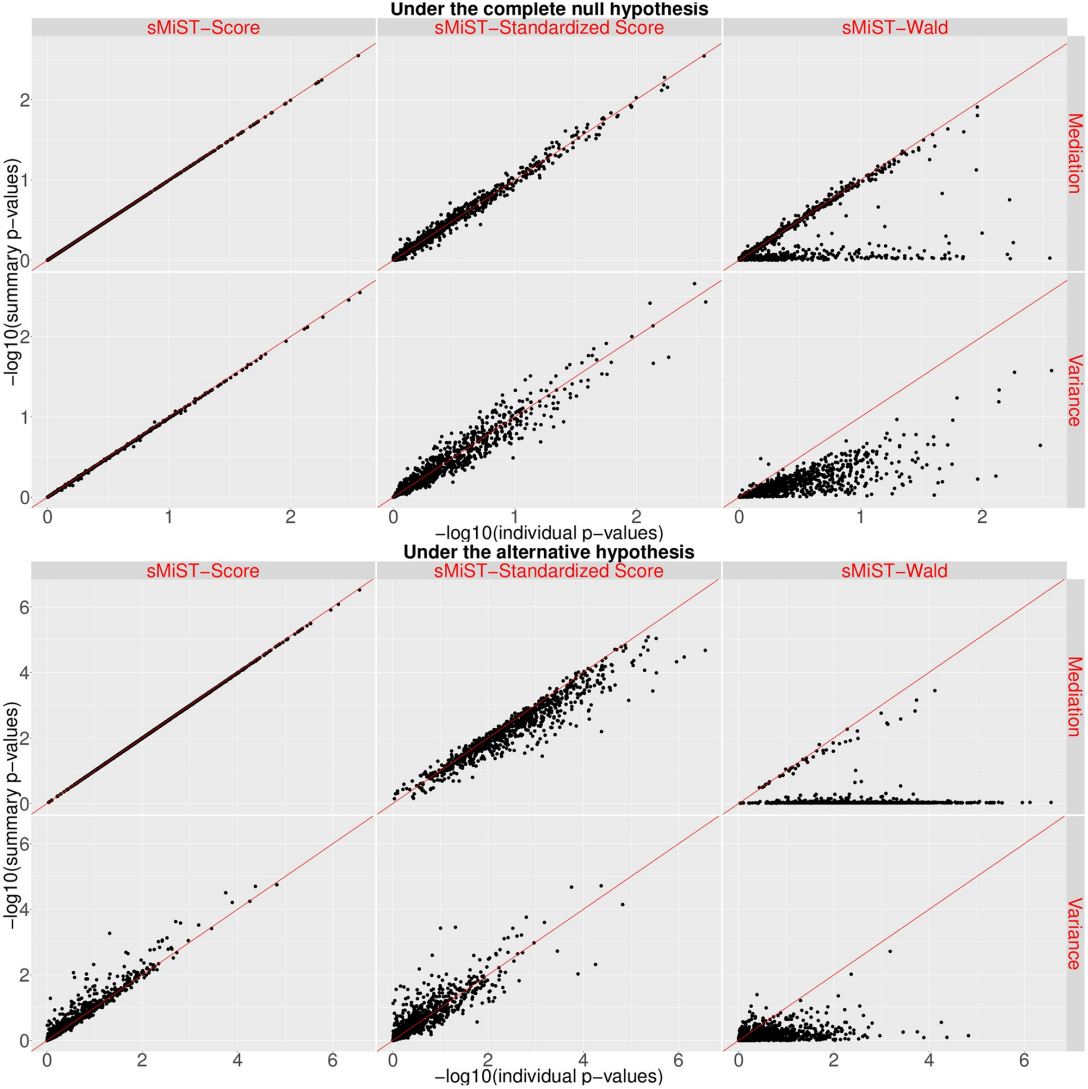
Comparison of -log10(p-values) from summary statistics based sMiST-Score, sMiST-Standardized Score, and sMiST-Wald vs. individual level data based MiST under the complete null hypothesis (top panel) and under the alternative hypothesis (bottom panel).

Fig 3 showed the comparison of these sMiST test statistics with MiST under the null and alternative hypothesis. It is clear that sMiST-Wald yields many outliers for both the mediation and variance components with p-values near 1 whereas the corresponding individual level data based MiST p-values range from 0 to 1. In contrast, the sMiST-Score agrees very well with MiST on both the mediation and variance component p-values under the null. Under the alternative, the mediation p-values still agree very well with MiST mediation p-values. For the variance component, while it generally agrees, sMiST-Score p-values are slightly inflated. sMiST-Standardized Score p-values also fall on the 45 degree line compared with MiST under the null and alternative; however, they have greater variation. Under the alternative, the p-values for mediation effects are slighly more conservative whereas the p-values for the variance component are slightly anti-conservative.

### Additional simulation results

We assessed the power of sMiST and its comparison with MiST under a wide range of scenarios: (1) varying strength of the association of G with M with *R*^2^ = 0.05, 0.2, and 0.8; (2) varying proportion of associated variants in the direct effects: Prop = 0.1, 0.2, 0.4, 0.6, and 0.8; and (3) mis-specification of the model for *M* given *G* where the true link function is log but the linear link is used to fit the model. As expected, as *R*^2^ increases, the power for the mediation effect increases, while the power for the direct effect stay the same (S5 Table). As the proportion of variants with direct effects increases, the power for mediation effect is constant but the power for the direct effects increases. As a result, the power for the total effect of mediation and direct effects increases under both scenarios.

When the relationship of G and M is mis-specified, the power for the mediation effect is reduced substantially when *R*^2^ = 0.05 but not as much when *R*^2^ = 0.2 and 0.8 (S6 Table). Interestingly, testing of direct effects can pick up some of the power loss for mediation effects due to model mis-specification. The power for the total effects under model misspecification is nearly the same as the power when the model is correctly specified when *R*^2^ ≥ 0.2 or the proportion of variants with direct effects ≥ 0.6.

### Performance of sMiST in real data analysis

An important input to sMiST or any summary statistics-based test statistics is the covariance matrix of the genetic variants. Instead of focusing on one or few genes, we evaluated the impact of the covariance matrix based on genome-wide real data analyses of GECCO studies for which we have individual level data for both outcomes and genotypes. Thus, we can directly compare the summary statistics-based test sMiST with the individual-level data based test MiST for a broad spectrum of genetic architecture and weight distribution in calculating predicted expression.

We obtained the summary statistics of marginal association log-odds ratio estimates and standard errors from GECCO data for sMiST to calculate the p-values for the mediator effect and variance component. In addition, using the individual level data, we obtained the mediation and variance component p-values using MiST, and treated these p-values as the gold standard for sMiST to be compared with.

**Fig 4:**
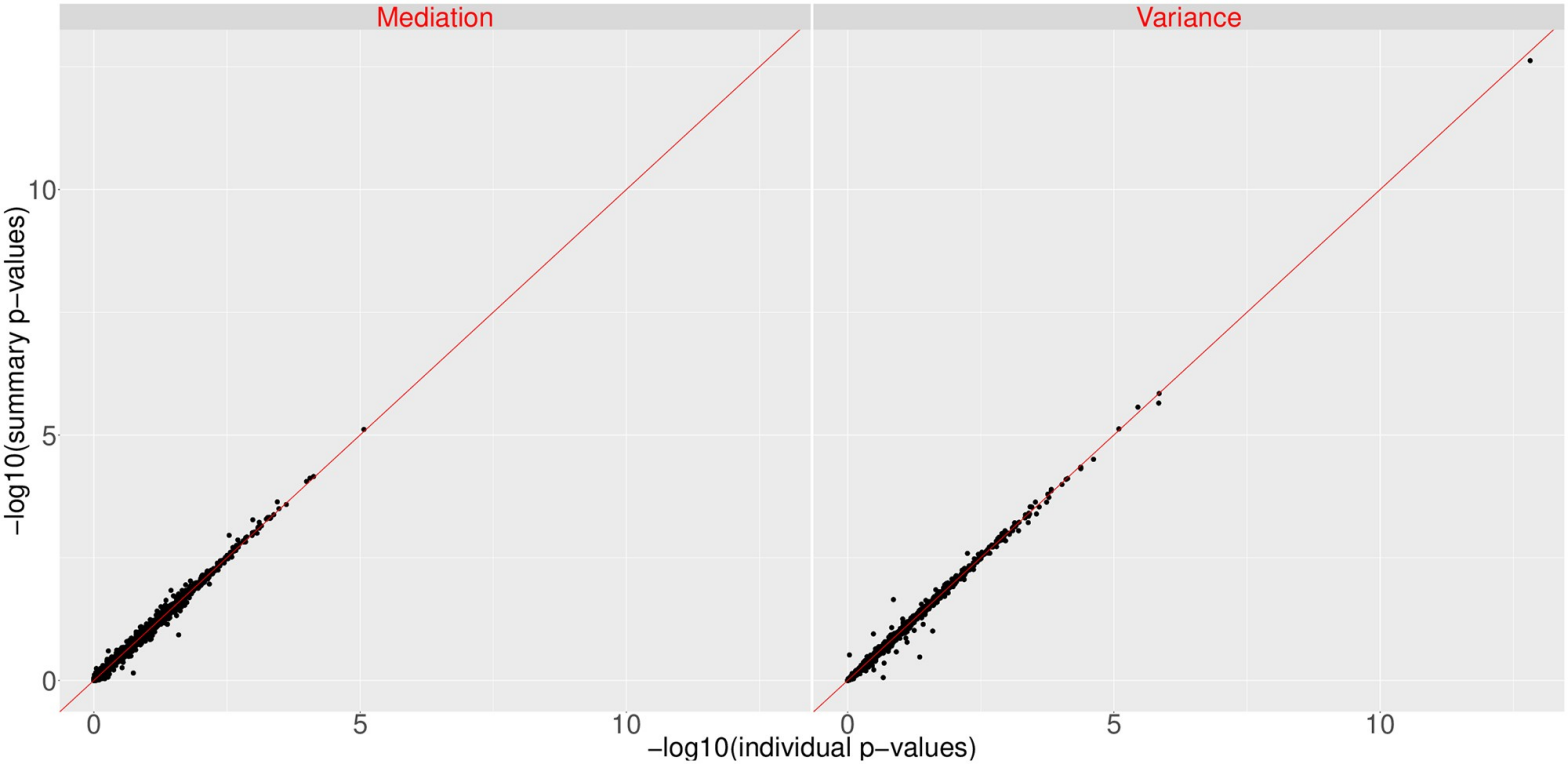
Comparison of sMiST using summary statistics from GECCO and LD matrices from CORECT with MiST with individual level data in GECCO.

In reality, the LD structure is often not available from the same source where the summary statistics are generated; hence external reference population are used to provide the estimated LD matrix on the variants. To evaluate our proposed method under such situation, we conducted a genome-wide analysis with summary level information from two different cohorts, GWAS summary statistics from GECCO and LD matrices calculated from CORECT. We compared the p-values of fixed effects and variance components from our method to those obtained from MiST based on individual level data in GECCO. From the scatter plots of the two sets of p-values as presented in Fig 4, we observe that the p-values on fixed effects and variance components from our method are comparable to the results from MiST using individual level data. The patterns of points aligning around the line of equality validate the proposed method under the situations when LD information from a similar external reference population is leveraged.

**Fig 5:**
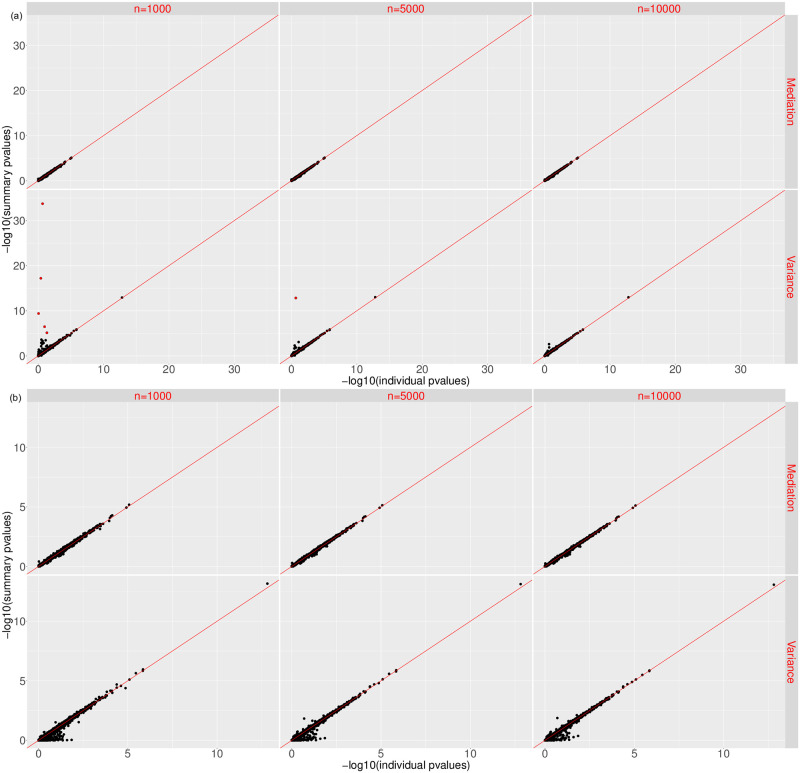
Effect of sample sizes in calculating the genotype covariance matrix on the mediation and variance component p-values for sMiST without regularization (top panel) and with regularization (bottom panel).

We then assessed the impact of the sample size of genotyping data needed for calculating the covariance matrix. We randomly sampled different sizes of sub-samples from GECCO and estimated the covariance of genotypes from the sub-samples. Fig 5 shows the scatter plots of −log10(p-values) of mediation and variance components obtained from sMiST with the covariance matrix based on *n* = 1, 000, 5, 000, and 10,000 samples, respectively, as compared with p-values from MiST. The p-values for sMiST and MiST generally fall on the 45 degree line; however, as the sample size becomes smaller, there are more and more outliers for the variance component test, where sMiST yields much smaller p-values compared to MiST. Upon close examination, these genes have an extreme correlation structure: all variants are in nearly perfect correlation with each other. For these extreme genes, the covariance estimates from small samples can be even more singular or perfectly singular. Although our method does not directly invert the whole covariance matrix for the mediation test, for the variance component testing it still involves inverting *W*^*T*^ cov(*G*)*W* while projecting out the mediation component. Therefore, in the situation of nearly singular matrix, it can be numerically unstable.

To avoid this numerical problem, we regularized the correlation matrix of *G* by adding λ*I*, where *I* is the identity matrix, as in the ridge regression. Following the asymptotic consistency results of Knight and Fu (2000) [17] for penalized regression, we chose λ based on sample size (*n*) used in calculating the covariance matrix: $\lambda =\frac{1}{\sqrt{n}\log\left(n\right)}$, such that the parameter estimates are consistent as *n* increases. We performed the regularization for all genes, since for a gene that is of moderation correlation structure, its covariance matrix is insensitive to the regularization. Fig 5b shows the scatter plots of regularized sMiST compared to MiST and it is clear that all outliers are gone even when *n* = 1, 000, and the regularization has minimal impact on the overall performance of sMiST. There are some points below the 45 degree line for the variance component test, suggesting sMiST may be slightly conservative. However, these generally occur when the p-values are large. When the p-values are small where they matter, sMiST even with regularization matches very well with MiST.

### Comparison of sMiST with S-PrediXcan and TWAS

**Fig 6:**
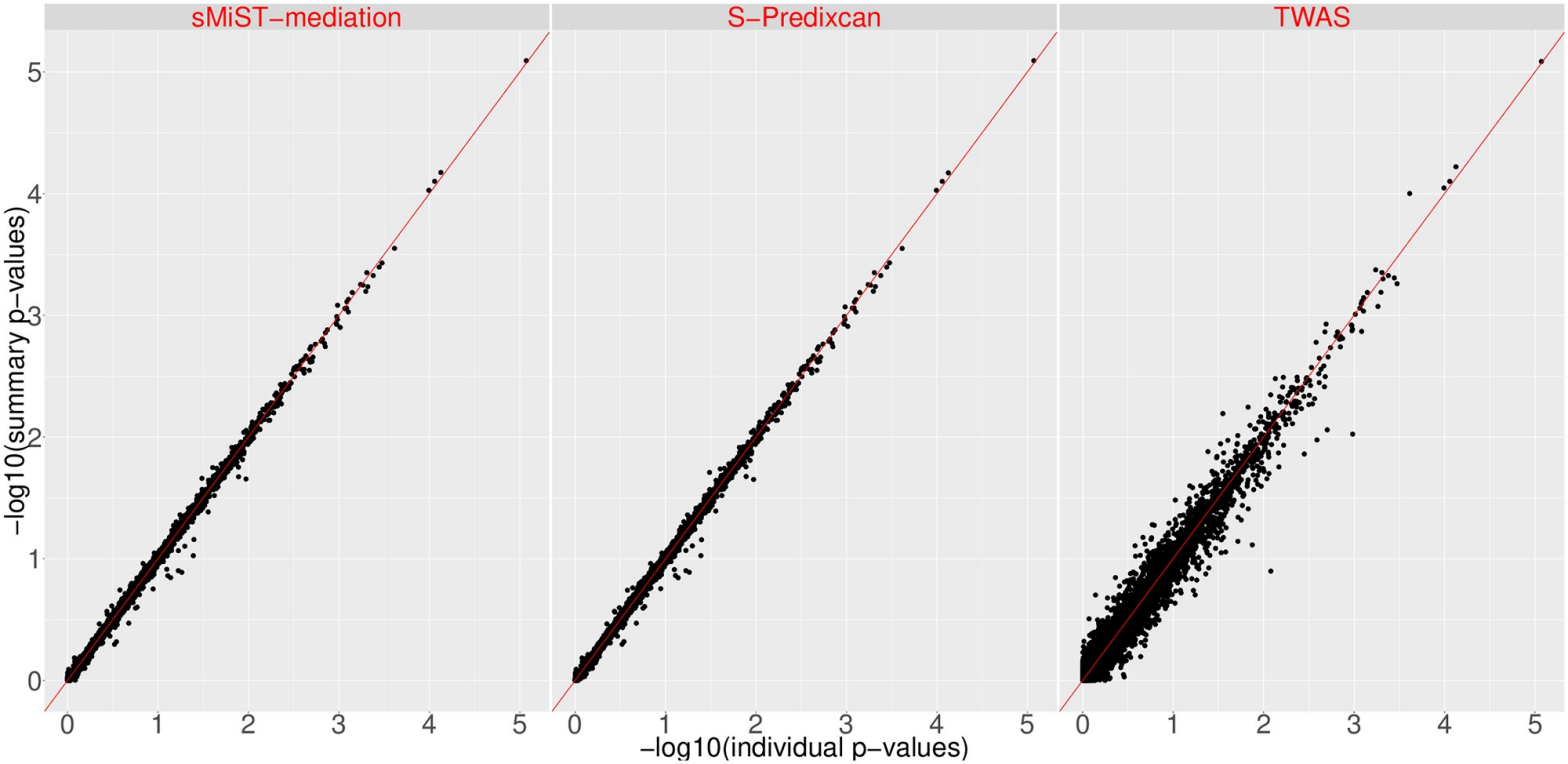
Scatter plots of −log10(p-values) from summary statistics-based methods sMiST mediation, S-PrediXcan, and TWAS vs. −log10(p-values) based on individual level data.

We compared the p-values for the predicted gene expression from sMiST (sMiST-mediation) as well as the two popular summary statistics-based methods S-PrediXcan [5] and TWAS [6] with the p-values calculated based on individual level data from GECCO, as described as in the previous section (Fig 6). The -log10(p-value)s fall around the 45 degree line for all methods, suggesting these summary statistics-based methods generally agree with individual level data-based p-values. Both sMiST and S-PrediXcan, which are nearly perfectly correlated with each other (S8 Fig), have a higher correlation with individual-level data based p-values than TWAS. Both sMiST and S-PrediXcan have the same estimator $\hat{\gamma }=W^{T}{\text{Cov}\left(G\right)W)}^{-1}W^{T}D{\hat{\beta }}^{*}$ but with slightly different variance estimator, where S-PrediXcan uses summary statistics, $\text{se}({\hat{\beta }}_{p}^{*})$ and the MAF of the *p*th SNP, to approximate the variance of the outcome while sMiST approximates the correlation of β* by the correlation matrix of genotypes. TWAS takes the weighted sum of Z statistics, which differs from S-PrediXcan and sMiST-mediation by a factor of the proportion of the phenotype explained by a SNP’s genotype [5, 6]. In general, this factor is close to 1; hence, we do not expect substantial difference between TWAS and S-PrediXcan and sMiST-mediation as shown in Fig 6.

## Discussion

We proposed a versatile set-based approach using summary statistics, sMiST, for testing the total effect of multiple mediators and direct effect. The computational time for sMiST is much faster than individual-level data based MiST. For example, for a dataset of 10,000 cases and 10,000 controls, MiST takes 0.955 seconds but sMiST takes only 0.022 seconds to calculate the p-value. sMiST also provides p-value for each mediator in the presence of other mediators under the assumption of *τ*^2^ = 0 and p-value for direct effects conditional on all the mediators. When there is evidence for mediator effect, we may further perform co-localization analysis using methods proposed previously [18, 19, 20, 21] to examine whether any specific genetic variant is pleiotropic to both the mediator and disease risk.

We offer a few observations from our extensive simulation and real data-based studies. Generally speaking, larger sample sizes lead to better estimates of the covariance matrix, and thus better alignment with results from individual level data. With growing external genotyping databases, having a large enough sample size to calculate the covariance is generally not a problem. To prevent numerical problems for genes with extreme correlation structures, we applied regularization for all genes, irrespective of correlation structures, in the hope that the regularization will minimize the numerical instability for genes with extreme correlation structures, while having minimal impact on other genes. Using this approach, we can mitigate bias that may arise due to high LD regions with a sample size as low as 1000. However, this regularization could potentially lower the power of our method, and yield slightly more conservative results. Such negative impact will be diminished, as our regularization is a function of sample size and it approaches 0 as the sample size increases.

Under all four scenarios, weaker correlation between confounder and fixed effects leads to better alignment between individual-level mediation effect p-values and summary-based mediation effect p-values, while the effect size of confounder does not affect the performance much. In particular, when the correlation is at the highest (0.4), summary statistics based mediation effect p-values can be somewhat over-conservative. The performance of the direct effect is not affected because of the orthogonalization of mediator and genotype in the data generation.

Generally, sMiST gains power by testing for the total association of mediation and direct effects compared to testing for only the mediation effect. However, when there is no direct effect, sMiST may lose some power due to testing an additional parameter of variance component that has null effect. More powerful combination methods can be employed to combine the test statistics for mediation and variance component to mitigate this impact [12]. These combination methods rely on only the p-values or test statistics and can be applied to sMiST. When the direct effect has a different sign than the mediated effect (inconsistent mediator) [22], the power for testing mediation effect and sMiST can be considerably reduced; however,if the direct effect is sufficiently strong, the power for mediation can approach to 1 (S7 Table). Under this situation, one needs to be cautious about the interpretation of mediation. Methods have been proposed to test the inconsistent mediation effect for one variable (here, one genetic variant) [23]. However, there lacks research for testing inconsistent mediation effect with multiple genetic variants. It is probably unlikely that a mediator is inconsistent with all genetic variants in practical situations. Nevertheless, it is a topic that warrants future research.

From our application of sMiST to CRC GWAS data, we identified three novel genes contributing to CRC risk that were not previously identified in the single variant analysis of the same dataset. *NT5DC2* has been shown to markedly reduce the expression of Fyn, a Src family proto-oncogene and has been implicated in glioblastoma [24], though not yet linked to CRC susceptibility. Of interest there are a couple of other nearby genes in the region, *NISCH* and *SEMA3G*, which share gene-linked regulatory elements, are expressed in T cells, and have been shown to play a role in CRC [25, 26]. For *VPREB3*, the protein encoded by this gene is thought to be involved in B-cell maturation, and may play a role in assembly of the pre-B cell receptor. A nearby gene, *CABIN1*, plays an important role in the T-cell receptor-mediated signal transduction pathway. Expression of this gene has previously been associated with CRC recurrence [27]. *PLD6* is a phospholipase of the outer mitochondrial membrane and acts as a regulator of mitochondrial shape by facilitating mitochondrial fusion [28]. Interestingly, a previous study showed that depletion of *PLD6* prevents *MYC* repression of *ANKRD1* and several other target oncogenes of YAP/TAZ. It has been hypothesized that mitochondrial dynamics, influenced in part by *PLD6*, might be an integral part of *MYC*-induced anabolic metabolism [29]. For *ANKRD10*, it is in a region dense with cancer-related genes (*CDKN2A* *CDKN2B*) and thus it is not surprising there may be multiple variants with independent regulatory effets. There is no report about the function of this gene; however, its paralog *ANKRD6* recruits CKI-epsilon to the beta-catenin degradation complex and allows efficient phosphorylation of beta-catenin, thereby inhibiting beta-catenin/Tcf signals [30]. As such, *ANKRD10* may have similar function in regulation of the Wnt pathway as *ANKRD6*.

It is of great interest to study the effect of interplay between mediation variables *M* and genetic variants *G* on the phenotypes. Huang et al. (2014) [14] derived *g*(*E*(*Y*|*G*, *X*) under the interaction model by integrating out *M* and found that the model depends not only on the linear terms of confounders *X* and *G*’s but also on the cross-product between *X* and *G*’s and the second order of *G*’s. Conceptually the proposed sMiST using summary statistics can be extended to study the interaction effect. However, the currently available summary statistics on marginal association do not permit modeling of interaction effects. Summary statistics on pairwise interaction among *G* as well as interaction between *X* and *G* will be needed in order to study the interaction effect of mediation.

## Methods and materials

### Ethics statement

This study uses the summary statistics of genome-wide association studies for colorectal cancer, and the de-identified genotyping data from GECCO, CCFR and CORECT. The study was approved by the Institutional Review Board at the Fred Hutchinson Cancer Research Center in Seattle, WA under file numbers 3995 and 6501.

### Derivation of sMiST

Assume that there are no confounders. We first focus on linear regression model. Consider a study of *n* independent individuals. Let *Y* be a *n* × 1 vector of outcomes, *G* a *n* × *P* matrix of *P* variants for the *n* individuals, *W* a *P* × *K* matrix with *W*_*pk*_ being the regression coefficient of *p*th variant for the *k*th mediator, and *D* is a diagonal matrix of cov(*G*). Further, let ${\hat{\beta }}^{*}={\left({\hat{\beta }}_{1}^{*},\ldots ,{\hat{\beta }}_{P}^{*}\right)}^{T}$. For simplicity, we center *G* and *Y* so that the intercept is 0. It is easy to see that

$$\begin{array}{c} n^{1/2}\left({\hat{\beta }}^{*}-\beta^{*}\right)={\left(\frac{D}{n}\right)}^{-1}n^{-1/2}{\left\{\sum_{i=1}^{n}G_{i1}\left(Y_{i}-G_{i1}\beta_{1}^{*}\right),\ldots ,\sum_{i=1}^{n}G_{iP}\left(Y_{i}-G_{iP}\beta_{P}^{*}\right)\right\}}^{T}, \end{array}$$

where β^*^ is the limit of ${\hat{\beta }}^{*}$. As *n* for GWAS typically is very large, under regularity conditions, by the continuous mapping theorem and the central limit theorem, $n^{1/2}\left({\hat{\beta }}^{*}-\beta^{*}\right)$ converges to a multivariate normal distribution with mean 0 and covariance of ${\hat{\beta }}^{*}$. Under *τ*^2^ = 0, the estimator for the mediation effect is

$$\begin{array}{ccc} \hat{\gamma } & = & {\left\{W^{T}\text{cov}\left(G\right)W\right\}}^{-1}W^{T}GY\\ & = & {\left\{W^{T}\text{cov}\left(G\right)W\right\}}^{-1}W^{T}D\left(D^{-1}GY\right)\\ & = & {\left\{W^{T}\text{cov}\left(G\right)W\right\}}^{-1}W^{T}D{\hat{\beta }}^{*}. \end{array}$$

We can obtain $\text{cov}(\hat{\gamma })$ as

$$\begin{array}{c} \text{cov}\left(\hat{\gamma }\right)={\left\{W^{T}\text{cov}\left(G\right)W\right\}}^{-1}W^{T}D\text{cov}\left({\hat{\beta }}^{*}\right)D^{T}W{\left\{W^{T}\text{cov}\left(G\right)W\right\}}^{-1}, \end{array}$$

where

$$\begin{array}{c} \text{cov}\left(\hat{\beta^{*}}\right)=[\begin{array}{ccc} \text{se}({\hat{\beta }}_{1}^{*}) & & \\ & \ddots & \\ & & \text{se}({\hat{\beta }}_{P}^{*}) \end{array}]\text{cor}\left(G\right)[\begin{array}{ccc} \text{se}({\hat{\beta }}_{1}^{*}) & & \\ & \ddots & \\ & & \text{se}({\hat{\beta }}_{P}^{*}) \end{array}]. \end{array}$$

Here cor(*G*) is the correlation matrix of *G*, which is the exact correlation of ${\hat{\beta }}^{*}$ under the null but an approximation under the alternative. Then, the test statistic for the mediation effect is

$$\begin{array}{c} U_{\gamma }={\hat{\gamma }}^{T}\text{cov}{\left(\hat{\gamma }\right)}^{-1}\hat{\gamma }. \end{array}$$

Under *H*_0_: *γ* = 0 and *τ*^2^ = 0, $U_{\gamma }∼\chi_{K}^{2}$. The test statistic for the *k*th mediator *γ*_*k*_ = 0 is ${\hat{\gamma }}_{k}/\text{se}\left({\hat{\gamma }}_{k}\right)∼N(0,1)$ for *k* = 1, …, *K*, where $\text{se}({\hat{\gamma }}_{k})$ is the square root of the *k*th diagonal element of $\text{cov}(\hat{\gamma })$.

For the variance component test, we derive the test statistic under *τ*^2^ = 0. By this, the variance component test adjusts for the mediator effect, and is independent of *U*_*γ*_ (Su et al. 2018) [12]. When combining the two test statistics using e.g., weighted linear combination, if they were correlated, the search space for the weight would be restricted. Independent test statistics can circumvent such restriction. Further, due to the non-conventional distribution for the variance component test, having independent test statistics can avoid the complex correlation structure and make it straightforward to derive the distribution of the combined test statistics. This is very useful, as it allows us to calculate p-values fast in a genome-wide search. Further, there are many methods to combine independent test statistics including popular p-values-based Fisher’s and Tippett’s combinations and data-adaptive combinations, which can be readily applied to our independent test statistics for mediation effect and variance component [12, 31].

The key to deriving the variance component test statistic conditioning on $\hat{M}$ is that, as opposed to using ${\hat{\beta }}_{p}^{*}$, we derive the summary statistics for each of *P* genetic variants ${\hat{\alpha }}_{p}^{*}$ by conditioning out ${\hat{M}}_{k},k=1,\ldots ,K$, which are given by

$$\begin{array}{c} {\hat{\alpha }}_{p}^{*}=A[\begin{array}{c} {\hat{\beta }}^{*}\\ {\hat{\beta }}_{p}^{*} \end{array}],\;\text{and}\;\;A=\left(0,0,...,0,1\right)C^{-1}[\begin{array}{cc} W^{T}D & 0\\ 0 & D_{p}\; \end{array}], \end{array}$$

where *D*_*p*_ is the *p*th diagonal entry of *D*, and *C* is a (*K* + 1) × (*K* + 1) matrix with $C_{jk}=W_{j}^{T}\text{cov}\left(G\right)W_{k}$ with *W*_*j*_ and *W*_*k*_ the *j*th and *k*th columns of *W*, and $C_{(K+1).}=C_{.(K+1)}^{T}=\left[\text{cov}{\left(G\right)}_{p.}W,D_{p}\right]$. The covariance of ${\hat{\alpha }}^{*}$ can then be straightforwardly obtained as $\text{cov}\left({\hat{\alpha }}^{*}\right)=A\text{cov}\left({\hat{\beta }}^{*}\right)A^{T}$. The test statistic for the variance component is

$$\begin{array}{ccc} U_{\tau^{2}} & = & U_{\alpha^{*}}^{T}U_{\alpha^{*}}, \end{array}$$

where $U_{\alpha^{*}}={\hat{\alpha }}^{*}/\text{var}\left({\hat{\alpha }}^{*}\right)$. Under the null, the variane component test *U*_*τ*^2^_ follows a mixture of $\chi_{1}^{2}$ with the mixture weighting as the eigenvalues of matrix *D*_α^*^_ R^*^ *D*_α^*^_, where *D*_α^*^_ is a diagonal matrix with $1/\text{se}({\hat{\alpha }}^{*})$, and R^*^ is the correlation matrix of ${\hat{\alpha }}^{*}$, both of which can be easily obtained from $\text{cov}({\hat{\alpha }}^{*})$.

Under the logistic regression model, by the Taylor’s expansion, we have $\hat{\gamma }-\gamma ={\left(W^{T}G^{T}\Delta GW\right)}^{-1}W^{T}G^{T}(Y-\mu )+o_{p}(n^{-1/2})$, where *Δ* is a diagonal matrix of *μ*(1 − *μ*) and *μ* = *E*(*Y*|*G*). Here *G* is centered. For simplicity of presentation, we omit any differences in the order of *o*_*p*_(*n*^−1/2^) because for the $\sqrt{n}$ asymptotic normality, these differences will be 0. Assume Δ is constant on the diagonal, we can reorganize $\hat{\gamma }-\gamma ={\left(W^{T}G^{T}GW\right)}^{-1}W^{T}D{\left(D\Delta \right)}^{-1}G^{T}\left(Y-\mu \right)={\left\{W^{T}\text{cov}\left(G\right)W\right\}}^{-1}W^{T}D\left({\hat{\beta }}^{*}-\beta^{*}\right)$. When the effects are modest, *γ* ≈ {*W*^*T*^ cov(*G*)*W*}^−1^ *W*^*T*^ Dβ^*^. As a result, $\hat{\gamma }={\left\{W^{T}\text{cov}\left(G\right)W\right\}}^{-1}W^{T}D{\hat{\beta }}^{*}$, which has the exactly same form as $\hat{\gamma }$ under the linear model. Under the null, $U_{\gamma }∼\chi_{K}^{2}$. We note that Δ is constant under the null. However, even when the null does not hold, Hu et al. (2013) [32] shows Δ does not strongly depend on covariates. Our extensive simulation also shows the proposed test statistics perform well under this approximation. Similar to the derivation for $\hat{\gamma }$, we can also obtain the test statistic for the variance component under the logistic regression model, which has the same form as the variance component test under the linear model.

When there are confounders, the derivation for test statistics using summary statistics becomes complicated. Under the liner model, $\hat{\gamma }$ is the weighted sum of *XY* and *W*^*T*^ *GY* with the weight as the corresponding elements in the inverse of the covariance matrix of (*X*, *W*^*T*^ *G*). If the effects of confounders are 0 or *X* and *G* are independent, then $\hat{\gamma }$ only depends on *G* and the above test statistics are the same. If the effects of confounders are not 0 and *X* and *G* are correlated, $\hat{\gamma }$ will depend on *X*; however, we observe our proposed test statistics hold very well based on our extensive simulations and real data analysis, suggesting that our test statistics are robust even in the presence of confounders.

### Datasets

Summary statistics (log-odds ratio estimates and standard errors of genome-wide genetic variants) were obtained from a meta-analysis of GWAS studies from three large consortia, including: the Genetics and Epidemiology of Colorectal Cancer Consortium (GECCO), the Colon Cancer Family Registry (CCFR), and the Colorectal Cancer Transdisciplinary Study (CORECT) [16]. In total, the consortia have 54,454 cases and 64,163 controls of European Ancestry. The genotyping data were imputed to the Haplotype Reference Consortium [33] with ∼40 million variants. The linkage disequilibrium or covariance of the genotypes was calculated using individual level data from GECCO (*n* = 26, 554). The details of study designs, genotyping QC, association and meta-analysis can be found elsewhere [16]. A brief summary of studies in these consortia is provided in S8 Table.

We downloaded the weights or regression coefficients of cis (< 1Mb from gene start or end) regulatory variants associated with gene expression for whole blood from the PredictDB Data Repository (http://predictdb.org/). The regression coefficients were estimated from a regularized linear regression model with elastic-net penalty [4]. The models were developed using a reference dataset of genotype and whole blood transcriptome data from 922 normal individuals from Depression Genes and Networks [34]. We considered genes of which the predictive *R*^2^ > 0.01 in the gene expression model, resulting in 8,893 genes. Using regulatory information derived from whole blood is relevant for studying susceptibility to CRC for two primary reasons. First, a subset of the immune-relevant cell types present in whole blood are relevant to CRC risk. In particular, T-cell populations of the intestine play a critical role in orchestrating the careful balance between immune activation and tolerance at the mucosal layer. Second, whole blood is the largest reference transcriptome dataset. As many tissues and cell types share common heritability in gene expression, in some cases whole blood models are preferred for building robust predictive models because of their large sample size.

### Performance of sMiST in simulation

We selected three genes (*CXCR1*, *C18orf32*, and *ARHGAP11A*) from the eQTL database from the PredictDB Data Repository. Both *CXCR1* and *C18orf32* are of moderate size (∼40 genetic variants), while *ARHGAP11A* is larger set (92 variants). In terms of the LD structure, both *C18orf32* and *ARHGAP11A* show largely independence or weak correlation among variants; however, *CXCR1* contains several clusters of variants that are nearly perfectly correlated.

We used the GWAS genotyping data from GECCO as the template (*n* = 26, 554), and generated the disease status under the generalized linear regression model (1) with logit link. We set the intercept to be −3, yielding about 5% baseline disease probability. We generated the mediator *M* = *cB* + *ϵ*, where $B=\sum_{p=1}^{P}w_{p}G_{p}$ was the genetically predicted gene, and *ϵ* ∼ *N*(0, *σ*^2^). Here, *c* and *σ*^2^ were set such that variation of *M* explained by *G* is 0.05, 0.20, and 0.80, while keeping the variance of *M* constant, which we set to be 1.5. The weights {*w*_*p*_, *p* = 1, …, *P*} were obtained from the PredictDB Data Repository. The effect of the mediator *M* was set to be log(2). Further, we let the random effects *δ*_*p*_ ∼ *N*(0, 0.05). To mimic the individual variant contributions that were not explained by predicted gene expression, we took the residuals from regressing the sum of direct effect $\sum_{p=1}^{P}\delta_{p}I_{p}G_{p}$ on *B* where *I*_*p*_ is 1 if *p*th variant has a direct effect and 0 otherwise, and added the residuals as direct effects to the model. The proportion of variants with direct effects was set to be 0.1, 0.2, 0.4, 0.6, 0.8, and 1.0. To save space, for most simulations presented in the main text, we set *R*^2^ = 0.05 and all variants have direct effects unless otherwise noted. Results for other parameter settings are provided in S4–S7 Tables. For each simulation setting, we generated 1000 simulated data sets, each set consisting of 1000 cases and 1000 controls.

## Implementation

We implemented sMiST using R programming language. The software is available for download at https://research.fhcrc.org/hsu/en/software.html.

## Supporting information

S1 Fig Gene *NT5DC2*. (a) forest plot of marginal association of genetic variants in *NT5DC2*; (b) forest plot of conditional association adjusting for predicted gene expression; (c) pairwise linkage disequilibrium (LD) *R*^2^. The p-values < 0.05 are labeled on the left margin in (a) and (b). The weights used in calculating gene expression are labeled on the right margin in (b).
(EPS)

S2 Fig Gene *VPREB3*. (a) forest plot of marginal association of genetic variants in *VPREB3*; (b) forest plot of conditional association adjusting for predicted gene expression; (c) pairwise linkage disequilibrium (LD) *R*^2^. The p-values < 0.05 are labeled on the left margin in (a) and (b). The weights used in calculating gene expression are labeled on the right margin in (b).
(EPS)

S3 Fig Gene *PLD6*. (a) forest plot of marginal association of genetic variants in *PLD6*; (b) forest plot of conditional association adjusting for predicted gene expression; (c) pairwise linkage disequilibrium (LD) *R*^2^. The p-values < 0.05 are labeled on the left margin in (a) and (b). The weights used in calculating gene expression are labeled on the right margin in (b).
(EPS)

S4 Fig Gene *ANKRD10*. (a) forest plot of marginal association of genetic variants in *ANKRD10*; (b) forest plot of conditional association adjusting for predicted gene expression; (c) pairwise linkage disequilibrium (LD) *R*^2^. The p-values < 0.05 are labeled on the left margin in (a) and (b). The weights used in calculating gene expression are labeled on the right margin in (b).
(EPS)

S5 Fig Scatter plots of -log10(p-values) of sMiST (Y-axis) and MiST (X-axis) for (a) mediation and (b) variance component for gene *C18orf32* under various confounding situations. (EPS)

S6 Fig Scatter plots of -log10(p-values) of sMiST (Y-axis) and MiST (X-axis) for (a) mediation and (b) variance component for gene *ARHGAP11A* under various confounding situations. (EPS)

S7 Fig Scatter plots of -log10(p-values) of sMiST (Y-axis) and MiST (X-axis) for mediation and variance component for gene *CXCR1* under various confounding situations. (EPS)

S8 Fig Pairwise comparison of −log10(p-values) between sMiST-mediation, S-PrediXcan, and TWAS. (EPS)

S1 Table List of significant genes associated with CRC risk. (PDF)

S2 Table Summary of OR (odds ratio) estimate, 95% CI (confidence interval) and p-value of predicted gene expression for the novel loci. (PDF)

S3 Table Sequential Analysis Results of the novel loci. (PDF)

S4 Table Type 1 error of sMiST and MiST with varying *R*^2^ and proportion of variants with direct effects (Prop) for gene *CXCR1*. (PDF)

S5 Table Power performance of sMiST vs. MiST with varying *R*^2^ and proportion of variants with direct effects (Prop) for gene *CXCR1*. (PDF)

S6 Table Power performance of sMiST vs. MiST under model misspecification with varying *R*^2^ and proportion of variants with direct effects for gene *CXCR1*. (PDF)

S7 Table Power performance of sMiST vs. MiST under inconsistent mediator, when *R*^2^ = 0.05 and the proportion of variants with direct effects is 0.80 for gene *CXCR1*. (PDF)

S8 Table Summary of study characteristics for the transcriptome-wide analysis of colorectal cancer. (PDF)
