# Supplementary material for: A general framework for functionally informed set-based analysis: Application to a large-scale colorectal cancer study
Source: PLoS Genet. 2020 Aug 24;16(8):e1008947. doi: 10.1371/journal.pgen.1008947 (PMC7470748; doi:10.1371/journal.pgen.1008947)
Supplement: S1 Table — (PDF) [file pgen.1008947.s009.pdf]

**Table S1.** List of significant genes associated with CRC risk at the significance level is $0.05/8893 = 4.5 \times 10^{-6}$. Unadjusted p-values and adjusted p-values are without and with adjusting for 140 CRC known loci. Pred. Gene is the p-value for predicted gene expression, Random is the p-value for the variance component, and oMiST is optimally linear weighted MiST combining Pred. Gene and Random p-vlaues.

| **Gene Info** | | | | **Unadjusted P-values** | | | **Adjusted P-values** | | |
|---|---|---|---|---|---|---|---|---|---|
| Gene | R2 | N.snps | Chrom | Pred.Gene | Random | oMiST | Pred.Gene | Random | oMiST |
| FHL3 | 0.73 | 37 | 1 | 1.30e-06 | 0.94 | 2.73e-06 | 0.90 | 0.93 | 0.99 |
| LAMC1 | 0.23 | 13 | 1 | 2.42e-16 | 0.86 | 2.89e-10 | 0.14 | 0.55 | 0.26 |
| SMG7 | 0.19 | 30 | 1 | 0.26 | 2.45e-08 | 5.02e-08 | 0.96 | 0.07 | 0.13 |
| TSEN15 | 0.15 | 59 | 1 | 0.50 | 1.36e-07 | 2.81e-07 | 0.48 | 0.39 | 0.64 |
| ARPC5 | 0.12 | 66 | 1 | 0.25 | 2.35e-16 | 2.95e-10 | 0.52 | 0.14 | 0.27 |
| HHIPL2 | 0.10 | 94 | 1 | 0.33 | 5.24e-07 | 1.07e-06 | 0.28 | 2.70e-03 | 5.66e-03 |
| NPL | 0.03 | 83 | 1 | 0.12 | 1.33e-09 | 1.94e-09 | 0.36 | 0.23 | 0.41 |
| C2orf62 | 0.40 | 18 | 2 | 0.17 | 2.50e-08 | 4.68e-08 | 0.03 | 0.40 | 0.07 |
| PNKD | 0.28 | 22 | 2 | 4.96e-08 | 5.41e-04 | 1.07e-07 | 0.34 | 8.23e-03 | 0.02 |
| CXCR2 | 0.15 | 32 | 2 | 5.15e-07 | 2.22e-03 | 1.07e-06 | 0.68 | 0.46 | 0.72 |
| TMBIM1 | 0.05 | 12 | 2 | 2.22e-04 | 1.95e-05 | 1.83e-06 | 0.42 | 0.71 | 0.66 |
| GPBAR1 | 0.05 | 20 | 2 | 1.23e-09 | 0.37 | 2.05e-09 | 0.96 | 0.84 | 0.97 |
| ARPC2 | 0.03 | 13 | 2 | 2.83e-07 | 0.63 | 7.36e-08 | 0.76 | 0.71 | 0.92 |
| SLC35A5 | 0.24 | 40 | 3 | 0.92 | 1.55e-06 | 3.51e-06 | 0.91 | 0.41 | 0.65 |
| PTGER4 | 0.09 | 48 | 5 | 0.91 | 2.36e-12 | 8.24e-11 | 0.48 | 0.07 | 0.14 |
| AHRR | 0.08 | 44 | 5 | 0.09 | 1.83e-07 | 2.86e-07 | 0.04 | 0.34 | 0.08 |
| HLA-C | 0.66 | 93 | 6 | 0.35 | 1.70e-07 | 3.43e-07 | 0.33 | 0.51 | 0.55 |
| HLA-B | 0.54 | 66 | 6 | 0.46 | 6.97e-14 | 1.41e-10 | 0.86 | 0.03 | 0.06 |
| MICB | 0.53 | 126 | 6 | 0.13 | 3.38e-08 | 6.49e-08 | 0.75 | 0.38 | 0.62 |
| HCG27 | 0.37 | 79 | 6 | 0.93 | 8.28e-09 | 1.66e-08 | 0.95 | 0.39 | 0.63 |
| LY6G5C | 0.28 | 60 | 6 | 0.55 | 5.20e-07 | 1.06e-06 | 0.53 | 0.73 | 0.79 |
| ATP6V1G2 | 0.25 | 92 | 6 | 0.24 | 9.23e-08 | 1.84e-07 | 0.94 | 0.31 | 0.53 |
| HSPA1B | 0.19 | 97 | 6 | 0.05 | 1.11e-06 | 1.97e-06 | 0.20 | 0.37 | 0.36 |
| LY6G5B | 0.16 | 32 | 6 | 0.06 | 4.03e-08 | 6.12e-08 | 0.68 | 0.29 | 0.49 |
| DDR1 | 0.14 | 47 | 6 | 0.08 | 1.07e-07 | 1.84e-07 | 0.90 | 0.64 | 0.88 |
| MSH5 | 0.13 | 39 | 6 | 0.60 | 5.53e-09 | 1.11e-08 | 0.33 | 0.43 | 0.56 |
| NOTCH4 | 0.13 | 59 | 6 | 0.76 | 1.64e-06 | 3.42e-06 | 0.55 | 0.19 | 0.34 |
| C6orf48 | 0.11 | 25 | 6 | 0.07 | 2.28e-06 | 3.37e-06 | 0.60 | 0.21 | 0.37 |
| LTA | 0.10 | 11 | 6 | 6.46e-08 | 1.46e-05 | 1.89e-08 | 0.27 | 0.51 | 0.46 |
| GTF2H4 | 0.10 | 66 | 6 | 1.70e-04 | 1.65e-06 | 1.40e-06 | 0.06 | 0.19 | 0.12 |
| FLOT1 | 0.10 | 60 | 6 | 0.06 | 1.09e-08 | 1.77e-08 | 0.30 | 0.26 | 0.46 |
| C2 | 0.09 | 41 | 6 | 5.88e-08 | 9.12e-04 | 1.23e-07 | 0.01 | 0.43 | 0.03 |
| TNF | 0.09 | 13 | 6 | 5.50e-09 | 0.59 | 1.07e-08 | 0.23 | 0.87 | 0.42 |
| GPANK1 | 0.09 | 57 | 6 | 0.81 | 1.37e-11 | 5.07e-11 | 0.28 | 0.57 | 0.48 |
| HSPA1L | 0.06 | 10 | 6 | 0.44 | 3.89e-07 | 8.69e-07 | 0.98 | 0.80 | 0.96 |
| DOM3Z | 0.04 | 37 | 6 | 0.81 | 1.38e-06 | 2.88e-06 | 0.86 | 0.83 | 0.97 |
| CSNK2B | 0.03 | 19 | 6 | 1.74e-03 | 2.82e-07 | 7.50e-08 | 0.34 | 0.48 | 0.57 |
| STK19 | 0.02 | 57 | 6 | 0.26 | 6.50e-08 | 1.31e-07 | 0.01 | 0.06 | 0.03 |
| C6orf136 | 0.01 | 15 | 6 | 0.97 | 4.58e-12 | 7.21e-11 | 0.94 | 0.10 | 0.19 |
| FAM84B | 0.21 | 30 | 8 | 0.07 | 2.59e-13 | 1.27e-10 | 0.42 | 0.18 | 0.32 |
| POU5F1B | 0.08 | 45 | 8 | 8.99e-08 | 2.01e-50 | 1.28e-07 | 0.04 | 0.06 | 0.09 |
| UTP23 | 0.02 | 21 | 8 | 3.70e-07 | 5.20e-08 | 8.97e-11 | 3.04e-03 | 0.55 | 7.41e-03 |
| C10orf57 | 0.17 | 126 | 10 | 0.59 | 4.71e-07 | 9.62e-07 | 0.98 | 0.19 | 0.34 |
| KIN | 0.11 | 31 | 10 | 0.03 | 3.05e-07 | 2.77e-07 | 0.07 | 0.16 | 0.15 |
| COX15 | 0.11 | 49 | 10 | 2.38e-07 | 3.48e-03 | 4.89e-07 | 0.59 | 0.34 | 0.57 |
| XRRA1 | 0.90 | 39 | 11 | 0.14 | 2.30e-08 | 4.12e-08 | 0.24 | 0.06 | 0.13 |
| PAAF1 | 0.52 | 115 | 11 | 0.35 | 1.73e-08 | 3.51e-08 | 0.39 | 0.07 | 0.13 |
| C11orf92 | 0.42 | 21 | 11 | 4.50e-04 | 2.78e-34 | 1.15e-08 | 0.30 | 0.31 | 0.51 |
| FADS1 | 0.24 | 13 | 11 | 3.31e-04 | 2.77e-05 | 9.81e-07 | 1.89e-03 | 0.30 | 4.77e-03 |
| KCNE3 | 0.16 | 42 | 11 | 0.88 | 1.47e-27 | 3.06e-09 | 0.75 | 0.08 | 0.16 |

| Gene Info | | | | Unadjusted P-values | | | Adjusted P-values | | |
|---|---|---|---|---|---|---|---|---|---|
| Gene | R2 | N.snps | Chrom | Pred.Gene | Random | oMiST | Pred.Gene | Random | oMiST |
| C11orf10 | 0.08 | 19 | 11 | 1.01e-06 | 0.23 | 2.08e-06 | 0.25 | 0.15 | 0.27 |
| PGM2L1 | 0.02 | 19 | 11 | 5.79e-05 | 9.28e-05 | 3.51e-06 | 0.04 | 0.50 | 0.08 |
| POLD3 | 0.01 | 6 | 11 | 7.82e-11 | 3.97e-06 | 1.81e-10 | 0.02 | 0.06 | 0.03 |
| DIP2B | 0.55 | 7 | 12 | 4.57e-12 | 0.01 | 1.27e-10 | 0.17 | 0.40 | 0.32 |
| TMEM116 | 0.17 | 30 | 12 | 0.01 | 8.24e-10 | 3.86e-11 | 0.43 | 0.23 | 0.42 |
| LIMA1 | 0.15 | 31 | 12 | 1.02e-11 | 0.04 | 5.19e-11 | 0.30 | 0.99 | 0.52 |
| ATF1 | 0.15 | 16 | 12 | 2.19e-04 | 3.33e-12 | 1.28e-10 | 0.74 | 0.14 | 0.27 |
| DYRK4 | 0.08 | 27 | 12 | 0.51 | 1.10e-11 | 5.22e-11 | 0.63 | 0.34 | 0.56 |
| ALDH2 | 0.07 | 24 | 12 | 0.10 | 7.47e-08 | 1.28e-07 | 0.08 | 0.06 | 0.12 |
| BCDIN3D | 0.06 | 51 | 12 | 0.40 | 3.86e-07 | 7.87e-07 | 0.48 | 0.19 | 0.34 |
| METTL7A | 0.05 | 40 | 12 | 6.90e-09 | 8.57e-03 | 1.33e-08 | 0.88 | 0.83 | 0.97 |
| SH2B3 | 0.04 | 26 | 12 | 6.91e-07 | 1.51e-03 | 1.47e-06 | 0.95 | 0.77 | 0.95 |
| NAA25 | 0.03 | 17 | 12 | 8.86e-07 | 0.65 | 1.92e-06 | 0.01 | 0.07 | 0.03 |
| PRPF40B | 0.02 | 64 | 12 | 0.18 | 6.07e-07 | 1.17e-06 | 0.85 | 0.01 | 0.03 |
| CCND2 | 0.01 | 14 | 12 | 0.05 | 4.64e-08 | 5.79e-08 | 0.95 | 0.35 | 0.58 |
| TCTN1 | 0.01 | 15 | 12 | 0.65 | 1.30e-06 | 2.83e-06 | 0.21 | 8.69e-03 | 0.02 |
| SMARCD1 | 0.01 | 7 | 12 | 3.85e-06 | 3.78e-09 | 5.67e-11 | 0.35 | 0.51 | 0.58 |
| BMP4 | 0.12 | 27 | 14 | 0.05 | 7.82e-08 | 1.13e-07 | 0.67 | 0.14 | 0.26 |
| ARHGAP11A | 0.26 | 92 | 15 | 0.92 | 4.45e-19 | 5.72e-10 | 0.35 | 0.11 | 0.21 |
| RPH3AL | 0.37 | 110 | 17 | 0.37 | 2.39e-07 | 4.93e-07 | 0.56 | 0.46 | 0.70 |
| FAM101B | 0.07 | 15 | 17 | 0.10 | 1.65e-07 | 2.70e-07 | 0.21 | 0.60 | 0.38 |
| GLOD4 | 0.03 | 52 | 17 | 0.98 | 8.32e-15 | 1.87e-10 | 0.23 | 0.45 | 0.41 |
| CRK | 0.02 | 34 | 17 | 0.18 | 8.24e-07 | 1.60e-06 | 0.90 | 0.42 | 0.66 |
| RPL17 | 0.11 | 51 | 18 | 0.21 | 3.67e-21 | 9.34e-10 | 0.82 | 0.03 | 0.06 |
| C18orf32 | 0.06 | 38 | 18 | 0.05 | 1.36e-29 | 4.64e-09 | 0.03 | 0.13 | 0.06 |
| SMAD7 | 0.03 | 19 | 18 | 9.04e-12 | 6.97e-21 | 4.58e-09 | 0.02 | 0.96 | 0.04 |
| GPATCH1 | 0.26 | 57 | 19 | 3.55e-07 | 4.38e-03 | 7.31e-07 | 0.01 | 0.07 | 0.02 |
| COL9A3 | 0.65 | 42 | 20 | 0.25 | 6.82e-08 | 1.42e-07 | 0.30 | 0.37 | 0.51 |
| TMX4 | 0.48 | 99 | 20 | 0.52 | 1.01e-12 | 9.57e-11 | 0.36 | 0.33 | 0.55 |
| LAMA5 | 0.17 | 11 | 20 | 9.19e-04 | 5.68e-08 | 4.02e-09 | 0.62 | 0.48 | 0.73 |
| CABLES2 | 0.13 | 41 | 20 | 0.44 | 2.65e-07 | 5.48e-07 | 0.45 | 0.55 | 0.71 |
| FAM65C | 0.13 | 24 | 20 | 0.02 | 7.38e-07 | 5.57e-07 | 0.64 | 0.07 | 0.15 |
| BMP2 | 0.13 | 41 | 20 | 5.64e-09 | 4.43e-17 | 6.81e-10 | 0.86 | 0.15 | 0.28 |
| CRLS1 | 0.13 | 40 | 20 | 0.60 | 1.22e-22 | 1.23e-09 | 0.54 | 0.51 | 0.76 |
| TRMT6 | 0.09 | 25 | 20 | 0.06 | 3.29e-09 | 4.88e-09 | 0.36 | 0.37 | 0.59 |
| PTK6 | 0.05 | 30 | 20 | 0.21 | 4.25e-07 | 8.54e-07 | 0.72 | 0.02 | 0.04 |
