# Supplementary material for: A general framework for functionally informed set-based analysis: Application to a large-scale colorectal cancer study
Source: PLoS Genet. 2020 Aug 24;16(8):e1008947. doi: 10.1371/journal.pgen.1008947 (PMC7470748; doi:10.1371/journal.pgen.1008947)
Supplement: S2 Table — (PDF) [file pgen.1008947.s010.pdf]

Table S2. Summary of OR (odds ratio) estimate, 95% CI (confidence interval) and p-value of predicted gene expression for the three novel loci (*NT5DC2, PLD6, VPREB3, VPREB*) and 1 novel secondary locus (*ANKRD10*).

**Novel genes: No known loci within 1Mb**									
**Gene Info**				**Unadjusted**			**Adjusted**		
Gene	R^2	N SNPs	chr	OR	95% CI	p-value	OR	95% CI	p-value
NT5DC2	0.35	52	3	1.00	[0.97,1.03]	0.96	1.00	[0.97,1.05]	0.95
VPREB3	0.04	12	22	1.00	[0.85,1.17]	0.99	~	~	~
PLD6	0.25	36	17	1.03	[0.98,1.10]	0.25	1.03	[0.97,0.94]	0.29
**Novel secondary genes: $\geq$ 1 known loci within 1mb**									
**Gene Info**				**Unadjusted**			**Adjusted**		
genename	R^2	N SNPs	chr	OR	95% CI	p-value	OR	95% CI	p-value
ANKRD10	0.06	36	13	1.04	[0.94,1.15]	0.48	1.04	[0.94,1.04]	0.43
