# Supplementary material for: A general framework for functionally informed set-based analysis: Application to a large-scale colorectal cancer study
Source: PLoS Genet. 2020 Aug 24;16(8):e1008947. doi: 10.1371/journal.pgen.1008947 (PMC7470748; doi:10.1371/journal.pgen.1008947)
Supplement: S3 Table — (PDF) [file pgen.1008947.s011.pdf]

Table S3. Sequential Analysis Results of the 3 novel loci (*NT5DC2, PLD6, VPREB3, VPREB*) and 1 novel secondary locus (*ANKRD10*). "Pred.Exp p-value" is the p-value for predicted gene expression. "Random p-value" is the p-value for the variance component of the random effects, after adjusting for genetic variants in "SNPs adjusted sequentially".

SNPs adjusted sequentially	Pred.Exp p-value	Random p-value
NT5DC2	0.95	2.03e-06
3:52491447_C/A	0.40	3.12e-03
3:53039455_G/A	0.08	0.23
PLD6	0.29	9.12e-07
17:17140683_A/G	0.13	9.05e-04
17:17141330_T/G	0.13	8.92e-04
17:16883680_G/A	0.8	0.49
VPREB3	0.99	1.41e-06
22:24087319	0.43	1.70e-04
22:24183198	0.84	0.04
22:24898310	0.93	0.41
ANKRD10	0.43	2.39e-05
13:111559742_G/A	0.04	0.05
13:112295716_G/A	0.02	0.55
13:111860386_A/G	0.02	0.63
13:111549315_C/T	0.02	0.63
13:110970108_T/C	0.02	0.73
13:111220415_C/T	0.05	0.87
13:110908777_T/G	0.05	0.94
13:111549790_C/T	0.17	0.94
