# Supplementary material for: A general framework for functionally informed set-based analysis: Application to a large-scale colorectal cancer study
Source: PLoS Genet. 2020 Aug 24;16(8):e1008947. doi: 10.1371/journal.pgen.1008947 (PMC7470748; doi:10.1371/journal.pgen.1008947)
Supplement: S5 Table — (PDF) [file pgen.1008947.s013.pdf]

Table S5. Power performance of sMiST vs. MiST with varying R^2 and proportion of variants with direct effects (Prop)† for gene *CXCR1*.

		Mediation		Variance		Combined	
R^2	Prop	MiST	sMiST	MiST	sMiST	MiST	sMiST
Varying R^2							
0.050	0.400	0.167	0.167	0.534	0.530	0.536	0.534
0.200		0.689	0.689	0.502	0.501	0.826	0.822
0.800		0.770	0.770	0.520	0.512	0.878	0.877
Varying prop of variants with direct effects							
0.200	0.100	0.681	0.681	0.173	0.172	0.641	0.640
	0.200	0.711	0.712	0.306	0.300	0.739	0.739
	0.400	0.689	0.689	0.502	0.501	0.826	0.822
	0.600	0.678	0.680	0.737	0.732	0.909	0.907
	0.800	0.705	0.705	0.850	0.848	0.949	0.946

$^{\dagger}\gamma = 0.1, b = 1.7$
