# Supplementary material for: A general framework for functionally informed set-based analysis: Application to a large-scale colorectal cancer study
Source: PLoS Genet. 2020 Aug 24;16(8):e1008947. doi: 10.1371/journal.pgen.1008947 (PMC7470748; doi:10.1371/journal.pgen.1008947)
Supplement: S6 Table — (PDF) [file pgen.1008947.s014.pdf]

Table S6. Power performance of sMiST vs. MiST under model misspecification with varying R^2 and proportion of variants with direct effects† for gene *CXCR1*.

		Mediation		Variance		Combined	
R^2	Prop	True	Misspec	True	Misspec	True	Misspec
0.050	0.200	0.763	0.494	0.216	0.323	0.774	0.591
0.200	0.200	0.793	0.744	0.294	0.301	0.806	0.772
0.800	0.200	0.775	0.769	0.315	0.305	0.814	0.802
0.050	0.400	0.782	0.515	0.410	0.547	0.840	0.763
0.200	0.400	0.787	0.731	0.504	0.535	0.878	0.862
0.800	0.400	0.771	0.751	0.563	0.548	0.880	0.864
0.050	0.600	0.759	0.460	0.568	0.712	0.885	0.835
0.200	0.600	0.773	0.707	0.724	0.738	0.925	0.898
0.800	0.600	0.767	0.740	0.734	0.726	0.927	0.918
0.050	0.800	0.773	0.457	0.658	0.807	0.919	0.889
0.200	0.800	0.763	0.702	0.795	0.809	0.947	0.940
0.800	0.800	0.775	0.755	0.810	0.806	0.956	0.948

†$\gamma = 0.1, b = 1.7$
