# Supplementary material for: A general framework for functionally informed set-based analysis: Application to a large-scale colorectal cancer study
Source: PLoS Genet. 2020 Aug 24;16(8):e1008947. doi: 10.1371/journal.pgen.1008947 (PMC7470748; doi:10.1371/journal.pgen.1008947)
Supplement: S7 Table — (PDF) [file pgen.1008947.s015.pdf]

Table S7. Power performance of sMiST vs. MiST under inconsistent mediator, when $R^2 = 0.05$ and the proportion of variants with direct effects is 0.80 for gene *CXCR1*.

	Mediation		Variance		Combined	
Model	MiST	sMiST	MiST	sMiST	MiST	sMiST
$\gamma = 0.25, b = 0$	0.705	0.705	0.042	0.042	0.597	0.596
$\gamma = 0.25, b = -0.5 * \gamma * c$	0.238	0.238	0.045	0.045	0.182	0.181
$\gamma = 0.25, b = -2.5 * \gamma * c$	0.954	0.954	0.033	0.033	0.909	0.908
