# Supplementary material for: A general framework for functionally informed set-based analysis: Application to a large-scale colorectal cancer study
Source: PLoS Genet. 2020 Aug 24;16(8):e1008947. doi: 10.1371/journal.pgen.1008947 (PMC7470748; doi:10.1371/journal.pgen.1008947)
Supplement: S8 Table — (PDF) [file pgen.1008947.s016.pdf]

Table S8. Summary of study characteristics for the transcriptome-wide analysis of colorectal cancer*

Study Acronym	Study Name	Country	N total	N Cases (Ad)	N Fem (%)	Mean age(SD), years	N Controls	N Fem (%)	Mean age(SD), years
ASTERISK	Association Study Evaluating RISK for sporadic colorectal cancer	France	1839	892 (0)	340 (38.1)	F: 69.3 (10.8) M: 68.4 (9.4)	947	423 (44.7)	F: 62.7 (10.5) M: 61.3 (9.8)
ATBC	Alpha-Tocopherol, Beta Carotene Cancer Prevention Study	Finland	177	147 (0)	0 (0)	F: - M: 57.4 (4.9)	30	0 (0)	F: - M: 57.2 (4.4)
CCFR1	Colon Cancer Family Registry	USA, Canada, Australia	2014	1036 (0)	500 (48.3)	F: 50 (11.6) M: 52.7 (11.3)	978	509 (52)	F: 57.6 (11.3) M: 58.5 (10.4)
CCFR2	Colon Cancer Family Registry	USA, Canada, Australia	716	331 (0)	179 (54.1)	F: 50.5 (10.5) M: 52.5 (10.6)	385	209 (54.3)	F: 53.4 (10.9) M: 52.8 (11.7)
CCFR3	Colon Cancer Family Registry	USA, Canada, Australia	1851	1190 (0)	608 (51.1)	F: 54.5 (11.9) M: 53.1 (11.1)	661	358 (54.2)	F: 52.3 (11.9) M: 53.3 (11.3)
CCFR4	Colon Cancer Family Registry	USA, Canada, Australia	2124	1590 (0)	634 (39.9)	F: 51.8 (12.3) M: 56.8 (11.6)	534	247 (46.3)	F: 50.8 (13.2) M: 55.6 (13.5)
Colo2&3	Hawai'i Colorectal Cancer Studies 2&3	USA	211	87 (0)	40 (46)	F: 66.3 (12.0) M: 65.2 (11.5)	124	54 (43.5)	F: 65.6 (10.5) M: 64.5 (11.4)
ColoCare Heidelberg	ColoCare Consortium	Germany	223	187 (0)	63 (33.7)	F: 63.4 (10.8) M: 62 (12.2)	36	19 (52.8)	F: 52.9 (10.1) M: 59.4 (14.5)
ColoCare Seattle	ColoCare Consortium	USA	169	169 (0)	73 (43.2)	F: 55.9 (12.3) M: 57.1 (13.8)	0	-	F: - M: -
CPSII1	American Cancer Society Cancer Prevention Study II nested case-control study	USA	1076	540 (0)	268 (49.6)	F: 68.7 (5.6) M: 69 (5.2)	536	260 (48.5)	F: 68.2 (5.6) M: 69.1 (5.3)
CRCGEN	Colorectal Cancer Genetics & Genomics, Spanish study	Spain	1546	760 (0)	269 (35.4)	F: 66.5 (11.9) M: 67.6 (10.4)	786	366 (46.6)	F: 62.9 (12.6) M: 66.8 (10.6)
DACHS1	Darmkrebs: Chancen der Verhütung durch Screening	Germany	3409	1707 (0)	708 (41.5)	F: 69.1 (11.2) M: 67.7 (9.7)	1702	685 (40.2)	F: 69.0 (10.0) M: 68.7 (10.3)
DACHS2	Darmkrebs: Chancen der Verhütung durch Screening	Germany	1164	666 (0)	260 (39)	F: 69.7 (11.9) M: 68.2 (10.5)	498	175 (35.1)	F: 70.8 (10.3) M: 69.2 (9.4)
DALS1	Diet, Activity and Lifestyle Study	USA	1411	702 (0)	303 (43.2)	F: 64.5 (9.9) M: 63.4 (9.4)	709	309 (43.6)	F: 64.3 (9.9) M: 63.2 (9.9)
DALS2	Diet, Activity and Lifestyle Study	USA	863	402 (0)	190 (47.3)	F: 63.6 (10.5) M: 64.1 (9.9)	461	220 (47.7)	F: 64.3 (9.8) M: 64.3 (10.2)
ESTHER VERDI	Epidemiologische Studie zu Chancen der Verhütung, Früherkennung und optimierten Therapie chronischer Erkrankungen in der älteren Bevölkerung; Verlauf der diagnotischen Abklärung bei Krebspatienten	Germany	817	397 (0)	136 (34.3)	F: 65.5 (8.4) M: 64.8 (6.6)	420	147 (35)	F: 65.3 (7.1) M: 65.1 (5.9)
HPFS1	Health Professionals Follow-Up Study	USA	455	227 (0)	0 (0)	F: - M: 66.6 (8.7)	228	0 (0)	F: - M: 66.1 (8.9)
HPFS2	Health Professionals Follow-Up Study	USA	348	176 (0)	0 (0)	F: - M: 63.3 (8.5)	172	0 (0)	F: - M: 64.0 (8.8)

HPFS3_AD	Health Professionals Follow-Up Study	USA	655	312 (312)	0 (0)	F: - M: 61 (8.6)	343	0 (0)	F: - M: 60.4 (8.3)
Kentucky	Kentucky Case-Control Study	USA	2167	1035 (0)	524 (50.6)	F: 61.5 (10.4) M: 62.1 (10.0)	1132	573 (50.6)	F: 66.7 (6.6) M: 60.4 (9.2)
MCCS	Melbourne Collaborative Cohort Study	Australia	1343	709 (0)	343 (48.4)	F: 60.0 (7.9) M: 59.9 (7.2)	634	309 (48.7)	F: 60.3 (7.3) M: 60.5 (7.8)
MEC1	Multiethnic Cohort Study	USA	816	389 (0)	183 (47)	F: 62.6 (7.8) M: 62.3 (8.5)	427	201 (47.1)	F: 63.4 (7.8) M: 62.7 (8.4)
MECC1	Molecular Epidemiology of Colorectal Cancer Study	Israel	978	483 (0)	218 (45.1)	F: 71.3 (10.7) M: 73.2 (9.3)	495	233 (47.1)	F: 71.9 (10.8) M: 73.6 (9.4)
MECC2	Molecular Epidemiology of Colorectal Cancer Study	Israel	1901	1093 (0)	549 (50.2)	F: 71.1 (11.3) M: 72.3 (10.5)	808	402 (49.8)	F: 72.8 (11.4) M: 75.0 (10.4)
MECC3	Molecular Epidemiology of Colorectal Cancer Study	Israel	4380	2570 (0)	1175 (45.7)	F: 67.4 (12.8) M: 68.8 (12.2)	1810	840 (46.4)	F: 68.9 (12.8) M: 69.9 (11.7)
MSKCC	Memorial Sloan Kettering Cancer Center Cohort	USA	68	68 (0)	41 (60.3)	F: 59.7 (12.3) M: 61.7 (12.7)	0	-	F: - M: -
NFCCR	Newfoundland Case-Control Study	Canada	660	193 (0)	73 (37.8)	F: 60.1 (8.7) M: 62.6 (8.5)	467	197 (42.2)	F: 58.1 (8.5) M: 60.2 (9.2)
NGCCS	PopGen Biobank	Germany	1103	1103 (0)	482 (43.7)	F: 62.4 (9.8) M: 64.8 (8.2)	0	-	F: - M: -
NHS1	Nurses' Health Study	USA	1165	391 (0)	391 (100)	F: 60 (6.6) M: -	774	774 (100)	F: 60.0 (6.5) M: -
NHS2	Nurses' Health Study	USA	339	158 (0)	158 (100)	F: 58.5 (6.3) M: -	181	181 (100)	F: 59.5 (6.3) M: -
NHS3 AD	Nurses' Health Study	USA	1090	513 (513)	513 (100)	F: 56.9 (6.7) M: -	577	577 (100)	F: 57.1 (6.8) M: -
NHSII	Nurses' Health Study	USA	167	87 (0)	87 (100)	F: 36.9 (4.5) M: -	80	80 (100)	F: 37.2 (4.0) M: -
OFCCR	Ontario Familial Colorectal Cancer Registry	Canada	1116	594 (0)	352 (59.3)	F: 62.1 (7.7) M: 61.1 (8.2)	522	227 (43.5)	F: 62.3 (8.1) M: 63.0 (7.5)
PHS	Physicians' Health Study	USA	764	375 (0)	0 (0)	F: - M: 59.3 (9.1)	389	0 (0)	F: - M: 58.6 (8.9)
PLCO_1	Prostate, Lung, Colorectal, and Ovarian Cancer Screening Trial	USA	2496	524 (0)	228 (43.5)	F: 64.7 (5.3) M: 64.9 (5.0)	1972	436 (22.1)	F: 63.5 (5.0) M: 64.1 (5.1)
PLCO_2	Prostate, Lung, Colorectal, and Ovarian Cancer Screening Trial	USA	889	475 (0)	204 (42.9)	F: 63.8 (5.3) M: 63.6 (5.2)	414	175 (42.3)	F: 63.7 (5.1) M: 63.5 (5.3)
PMH SC-CFR	Postmenopausal Hormones Supplementary Study to Seattle Colon Cancer Family Registry	USA	398	276 (0)	276 (100)	F: 63.2 (6.8) M: -	122	122 (100)	F: 61.6 (7.4) M: -
SEARCH	Studies of Epidemiology and Risk Factors in Cancer Heredity	UK	4288	4173 (0)	1789 (42.9)	F: 62.7 (8.2) M: 63.8 (7.4)	115	46 (40)	F: 53.6 (7.3) M: 55.5 (7.0)
SLRCCS	Swedish Low-Risk Colorectal Cancer Study	Sweden	4785	2504 (0)	1135 (45.3)	F: 61.7 (0.0) M: 61.7 (0.0)	2281	1085 (47.6)	F: 61.7 (0.0) M: 61.7 (0.0)
SMC COSM	Swedish Mammography Cohort and Cohort of Swedish Men	Sweden	1397	566 (0)	235 (41.5)	F: 64.6 (9.0) M: 63.7 (8.2)	831	348 (41.9)	F: 63.7 (8.2) M: 63.8 (8.1)
USC HRT CRC	Los Angeles County Cancer Surveillance Program	USA	708	321 (0)	321 (100)	F: 66.4 (5.5) M: -	387	387 (100)	F: 64.9 (6.8) M: -
VITAL	VITamins And Lifestyle	USA	565	279 (0)	129 (46.2)	F: 66.7 (5.9) M: 65.9 (6.4)	286	138 (48.3)	F: 67.2 (6.1) M: 66.0 (6.4)
WHI1	Women's Health Initiative Study	USA	1991	468 (0)	468 (100)	F: 67.3 (6.8) M: -	1523	1523 (100)	F: 69.5 (6.4) M: -
WHI2	Women's Health Initiative Study	USA	1984	978 (0)	978 (100)	F: 66 (6.6) M: -	1006	1006 (100)	F: 65.6 (6.2) M: -
WHI3	Women's Health Initiative Study	USA	1113	554 (0)	554 (100)	F: 65.1 (6.5) M: -	559	559 (100)	F: 64.4 (6.3) M: -
CLUEII	Campaign against Cancer and Heart Disease II	USA	518	258 (0)	137 (53.1)	F: 62 (11.4) M: 61.0 (10.7)	260	141 (54.2)	F: 61.4 (11.9) M: 60.2 (10.7)

COLON	Colorectal Cancer: Longitudinal observational study on nutritional and lifestyle factors that influence colorectal tumor recurrence, survival and quality of life	Netherlands	1335	643 (0)	235 (36.5)	F: 64.3 (10.1) M: 65.8 (8.9)	692	256 (37)	F: 60.8 (6.9) M: 62.0 (6.2)
CORSA1	Colorectal Cancer Study of Austria	Austria	2234	1460 (519)	545 (37.3)	F: 65.1 (11.2) M: 64.1 (10.7)	774	332 (42.9)	F: 63.1 (10.9) M: 62.9 (10.1)
CORSA2	Colorectal Cancer Study of Austria	Austria	2483	1210 (687)	426 (35.2)	F: 65.9 (11.2) M: 65.8 (10.5)	1273	451 (35.4)	F: 60.5 (12.5) M: 55.5 (13.0)
CPSII2	American Cancer Society Cancer Prevention Study II nested case-control study	USA	688	339 (0)	176 (51.9)	F: 68.3 (6.0) M: 69.3 (5.1)	349	184 (52.7)	F: 68.1 (6.1) M: 69.2 (5.1)
Czech	Czech Republic CCS	Czech Republic	3293	1675 (0)	624 (37.3)	F: 63 (11.8) M: 63.7 (10.8)	1618	735 (45.4)	F: 50.3 (14.8) M: 50.8 (12.5)
DACHS3	Darmkrebs: Chancen der Verhütung durch Screening Study	Germany	1827	1210 (0)	456 (37.7)	F: 69.5 (11.3) M: 68.1 (10.4)	617	242 (39.2)	F: 65.8 (12.9) M: 67.2 (10.8)
EDRN	Early Detection Research Network	USA	589	273 (6)	107 (39.2)	F: 62.4 (13.4) M: 62.7 (11.6)	316	161 (50.9)	F: 59.1 (10.4) M: 60 (10.8)
EPIC	European Prospective Investigation into Cancer and Nutrition	Europe	4401	2095 (0)	1116 (53.3)	F: 56.9 (8.1) M: 57.2 (8.2)	2306	1232 (53.4)	F: 56.6 (8.1) M: 56.8 (8.1)
EPI COLON	EPICOLON	Spain	609	267 (0)	110 (41.2)	F: 54.1 (10.2) M: 57.4 (9.1)	342	141 (41.2)	F: 58.7 (6.6) M: 59.7 (6.4)
Hawaii CCS AD	Hawaii Adenoma Study	USA	628	85 (85)	36 (42.4)	F: 59.6 (9.1) M: 58.6 (8.0)	543	191 (35.2)	F: 60.4 (8.4) M: 59.0 (7.5)
HPFS4	Health Professionals Follow-Up Study	USA	380	183 (0)	0 (0)	F: - M: 72.4 (8.6)	197	0 (0)	F: - M: 72.0 (8.7)
HPFS5 AD	Health Professionals Follow-Up Study	USA	260	155 (155)	0 (0)	F: - M: 67.3 (7.9)	105	0 (0)	F: - M: 67.8 (7.9)
LCCS	Leeds Colorectal Cancer Study	UK	2183	1482 (0)	598 (40.4)	F: 69.7 (10.9) M: 68.7 (9.8)	701	371 (52.9)	F: 67.7 (9.1) M: 68.3 (8.1)
NCCCSI	North Carolina Colon Cancer Study, I	USA	720	251 (0)	111 (44.2)	F: 65.1 (10.1) M: 64.8 (9.5)	469	205 (43.7)	F: 66.1 (9.5) M: 66.0 (9.6)
NCCCSII	North Carolina Colon Cancer Study, II	USA	1281	595 (0)	241 (40.5)	F: 62.3 (10.4) M: 62.2 (10.4)	686	259 (37.8)	F: 63.3 (10.7) M: 64.6 (9.1)
NHS4	Nurses' Health Study	USA	611	308 (0)	308 (100)	F: 70.1 (9.3) M: -	303	303 (100)	F: 70.3 (9.0) M: -
NHS5 AD	Nurses' Health Study	USA	477	251 (251)	251 (100)	F: 67.3 (7.6) M: -	226	226 (100)	F: 67.6 (7.7) M: -
NSHDS	The Northern Sweden Health and Disease Study	Sweden	829	416 (0)	267 (64.2)	F: 57.5 (7.9) M: 53.3 (7.2)	413	265 (64.2)	F: 57.6 (7.9) M: 53.3 (7.4)
OSUMC	Columbus-area HNPCC study, Ohio Colorectal Cancer Prevention Initiative, Ohio State University Medical Center	USA	5527	3094 (0)	1428 (46.2)	F: 62.4 (13.2) M: 61.4 (12.9)	2433	1472 (60.5)	F: 51.1 (15.0) M: 53.3 (14.4)
PLCO4 AD	Prostate, Lung, Colorectal, and Ovarian Cancer Screening Trial	USA	2105	797 (794)	337 (42.3)	F: 62.9 (5.5) M: 62.5 (5.2)	1308	737 (56.3)	F: 61.9 (5.3) M: 61.9 (5.3)
SELECT	Selenium and Vitamin E Prevention Trial	USA	533	264 (0)	0 (0)	F: - M: 65.9 (6.3)	269	0 (0)	F: - M: 65.8 (6.6)
SMS AD	Screening Markers for Colorectal Cancer Study (advanced adenomas)	USA	171	41 (0)	16 (39)	F: 65.6 (10.7) M: 63.4 (9.3)	130	73 (56.2)	F: 58.9 (11.3) M: 62.5 (11.2)
UKB	UK Biobank	UK	26763	5356 (352)	2275 (42.5)	F: 61.2 (6.3) M: 62.1 (5.8)	21407	9084 (42.4)	F: 59.3 (7.2) M: 60.4 (6.7)

* The European ancestry set from the table is 118,617.
